# Reversible Carbon Dioxide Capture and Release using an Electropolymerized Anthraquinone Electrode in Aqueous Solution

**DOI:** 10.1021/acsami.5c17350

**Published:** 2025-10-08

**Authors:** Elisabeth Leeb, Dominik Wielend, Nadine Kleinbruckner, Daniel Werner, Corina Schimanofsky, Victoria Greussing, Katharina Matura, Engelbert Portenkirchner, Niyazi S. Sariciftci

**Affiliations:** 1 Linz Institute for Organic Solar Cells (LIOS), Institute of Physical Chemistry, 27266 Johannes Kepler University Linz, Altenberger Straße 69, Linz 4040, Austria; 2 Institute of Physical Chemistry, 27255 University of Innsbruck, Innrain 52c, Innsbruck 6020, Austria

**Keywords:** CO_2_ capture and release, redox-active polymers, electropolymerization, carbon-based electrodes, pH dependence, electrochemical impedance spectroscopy

## Abstract

The rise of carbon dioxide (CO_2_) in the atmosphere is closely linked to global climate change, driving the need for efficient carbon capture technologies. This study investigates the electrochemical carbon capture capabilities of the polymer poly-1-aminoanthraquinone (p-1-AAQ) when coated onto glassy carbon and carbon paper electrodes. This polymer is synthesized from cheap materials using facile, oxidative electropolymerization and provides high cyclic stability. Cyclic voltammetry and potentiostatic “electroswing” methods were employed together with infrared spectroscopy detection to study CO_2_ capture and evaluate Faradaic efficiency under acidic, neutral, and alkaline conditions. These results indicate that coated glassy carbon electrodes offer significantly higher Faradaic efficiencies than carbon paper (CP). However, carbon paper electrodes still displayed an exemplary maximum capture efficiency of 76%, showing that 2 CO_2_ molecules were captured per polymeric anthraquinone repeating unit. With a low loading of electroactive polymer on the overall electrode, a good CO_2_ uptake capacity of 0.17 mmol_CO2_ g_p‑1‑AAQ+CP_^–1^ based on the whole immersed electrode mass was achieved. Electrochemical impedance spectroscopy revealed that differences in interface resistance between the polymer and the electrolyte contribute to this disparity, particularly at lower potentials where glassy carbon shows suppressed unwanted side reactions.

## Graphical abstract

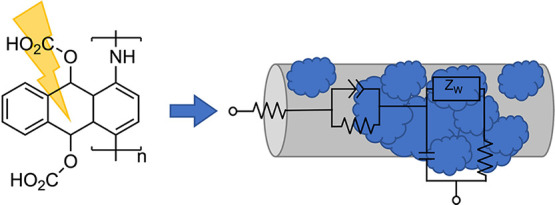

## 1 Introduction

The continuous rise in global energy demand has intensified the reliance on fossil fuels, which remain a predominant source of cheap and dependable energy. Consequently, anthropogenic carbon emissions have increased significantly, contributing to the growing concentration of greenhouse gases in the atmosphere.^1–3^ This trend poses a significant challenge to environmental sustainability, as it drives global temperature rise and threatens long-term prosperity and economic growth, particularly in disadvantaged regions worldwide.^4,5^

Expanding renewable energy sources, such as wind, solar, and hydropower, has been a primary focus over the past decade to counteract these effects. In addition, advancements in energy-efficient technologies aim to reduce carbon footprints. However, achieving a fully carbon-neutral society remains a challenging goal, as both the power and industrial sectors still heavily depend on fossil fuels. Carbon mitigation strategies, including carbon capture and storage (CCS) and carbon capture and utilization (CCU), offer promising avenues for addressing emissions from carbon-intensive point sources.^6,7^ These methods are particularly relevant for industrial processes that generate high concentrations of carbon dioxide (CO_2_) in flue gases. Traditionally, carbon capture involves passing exhaust gases through amine-based absorbent solutions, where CO_2_ is selectively absorbed at room temperature. Subsequent thermal regeneration releases CO_2_, allowing the absorbent to be reused in a cyclic process.^8–10^ Despite their efficacy, these methods are often energy-intensive and costly, primarily due to the need for thermal desorption.^11^

In contrast, electrochemical carbon capture offers an interesting alternative by utilizing electrical energy rather than heat for CO_2_ capture and release. This approach is more energy-efficient and can best be implemented using renewable electricity sources. Electrochemical methods typically involve applying a potential to facilitate carbon capture without the need for high temperatures.^12–14^ As a result, these systems offer a perspective to scalable, energy-efficient solutions that ease the transition toward the primary goal of a low-carbon economy. Although the scientific field of electrochemical CO_2_ capture methods is already well reviewed, a possible large-scale industrial application thereof still faces several problems in comparison to established technologies.^15^

The concept of electrochemical CO_2_ capture dates back to 1969 when Huebscher and Babinsky proposed a two-stage method involving initial gas capture followed by selective separation.^16^ Today, electrochemical carbon capture technologies fall into three main categories: pH-mediated systems, electrochemically mediated amine regeneration, and redox-active molecule systems. Among the most promising approaches are quinone-based electrochemical systems, which efficiently capture CO_2_ *via* reversible redox reactions, and methods employing transition metal complexes and pyridines to form stable carbon adducts.^17^ Transition metal complexes can directly bind CO_2_ through coordination chemistry.^18,19^ These systems benefit from tunable redox potentials and structural versatility.

Quinones are versatile redox-active molecules that undergo reversible two-electron reductions to form hydroquinones. These reduced quinone species can then further react with CO_2_, binding it *via* the reduced carbonyl moiety.^20–22^ While quinones are advantageous due to their wide availability and diverse derivatives, their limited solubility in aqueous solutions as well as the stability of the reduced quinone species remain challenging. Additionally, competing processes such as protonation and ion association hinder the reactivity of quinones toward CO_2_ capture in aqueous media.^23–25^ Notably, anthraquinone derivatives have also found significant applications as active materials in organic batteries, where their tunable redox properties and reversible electron-transfer characteristics support efficient and reliable energy storage.^26–28^

A further direction for electrochemical carbon capture lies in exploring novel, cost-effective, and resilient materials under operational conditions. Redox-active covalent organic frameworks^29–31^ and hybrid organic–inorganic materials^32,33^ are under development to enhance the capture and release efficiency. The ability to fine-tune redox properties and increase the durability of these materials will be crucial for real-world applications.

Various anthraquinones have also demonstrated a notable ability to enable CO_2_ capture, both when immobilized on electrode surfaces^34–36^ and homogeneously in solution.^37–40^ In particular, recent advances by Yang and co-workers have highlighted molecular design principles and mechanistic insights into electrochemical CO_2_ capture and redox-mediated separation.^17,41^ Additionally, Hatton and co-workers demonstrated the effectiveness of a polyanthraquinone-carbon nanotube composite in capturing CO_2_ *via* “electroswing” reactive absorption, showcasing the potential for efficient carbon capture under mild conditions.^42,43^ Building on these advancements upon immobilization and combining the beneficial effect of a hydrogen-bonding amino group from homogeneous studies,^39^ the present study investigates poly-1-aminoanthraquinone (p-1-AAQ), which has been synthesized through oxidative electrochemical polymerization. This approach bypasses the negative effects of the dissolution of soluble hydroquinone species as the resulting polymer is both reductively and oxidatively stable.^34^ Furthermore, the facile synthetic approach, utilizing relatively inexpensive educts, yields a conjugated polymer featuring a conductive anthraquinone-based backbone and amino moieties in close proximity to the carbonyl groups. Conjugated polymers, such as polyanthraquinones or polyaniline, are particularly attractive for electrochemical CO_2_ capture due to their delocalized π-systems, which facilitate efficient electron transfer processes. Moreover, their redox-active nature and structural modularity enable the fine-tuning of their electrochemical behavior.^44^

Herein, we focus on p-1-AAQ’s capabilities for heterogeneous carbon capture in aqueous environments under acidic, neutral, and alkaline conditions using two different carbon-based electrodes and two different methods of electrochemical carbon capture. In addition, electrochemical impedance spectroscopy (EIS) was employed to elucidate further the charge transfer mechanisms involved during carbon capture. This approach facilitates an understanding of the charge transfer relation between the polymer and the electrodes, as well as understanding the difference in faradaic efficiency when employing varying methods of carbon capture. Our results show an astounding molar uptake efficiency of 76%, demonstrating anthraquinone’s capability to capture 2 mol CO_2_ per mol repeating unit of the polymer.

## 2 Experimental Section

### 2.1 Electrode Preparation and Electrochemical Polymerization

Glassy carbon (GC) plate electrodes (1 × 4 cm, 2 mm, Alfa Aesar) were polished using Al_2_O_3_ pastes (Buehler Micropolish II deagglomerated) using decreasing particle sizes of 1.0, 0.3, and 0.05 μm. The electrodes were sonicated first in 18 MΩ water between each polishing step, followed by isopropanol. To finish this procedure, the electrodes were electrochemically activated by sweeping the potential between −0.85 and 1.65 V at a scan rate of 50 mV s^–1^ for 30 cycles in an electrolyte solution of 0.5 M H_2_SO_4_. Carbon paper electrodes (CP, TGP-H 60, Alfa Aesar) were cut into 1 × 4 cm pieces and contacted using a copper wire and small, silver-plated clamps.

P-1-AAQ was produced through oxidative, electrochemical polymerization, as described previously.^45,46^ A 5 mM solution of 1-aminoanthraquinone (1-AAQ, Alfa Aesar) in 6 M H_2_SO_4_ was purged with N_2_ for 30 min. Afterward, the potential was cycled between 1.46 and 0.16 V for 40 cycles at a scan rate of 100 mV s^–1^.

### 2.2 Electrochemical Experiments and Carbon Capture

Unless otherwise indicated, all potentials are recalculated against standard hydrogen electrode (SHE).^47^ All experiments were performed on an IPS Jaissle Potentiostat/Galvanostat PGU 10 V - 100 mA or a Jaissle Potentiostat/Galvanostat 1030 PC.

All experiments were performed in a one-compartment cell, with the respective modified, carbon-based electrode as the working electrode (WE), a platinum foil as the counter electrode (CE), and a commercial Ag/AgCl/3 M KCl electrode (Basi) as the reference electrode (RE). The RE was calibrated using potassium ferrocyanide (Merck). Half-step potentials (E_p/2_) were obtained by determining the potential corresponding to half of the peak current. Three different 0.1 M phosphate buffer solutions were used as electrolyte solutions. The buffer solutions were prepared from H_3_PO_4_ (Sigma-Aldrich, 85 wt % in H_2_O) and NaH_2_PO_4_ · 2 H_2_O (Sigma-Aldrich) for pH 2, NaH_2_PO_4_ · 2 H_2_O and Na_2_HPO_4_ (Sigma-Aldrich) for pH 7, and Na_2_HPO_4_ and Na_3_PO_4_ · 12 H_2_O (Thermo-Scientific) for pH 11.

The cell was flushed with N_2_ for 30 min prior to any experiments. Afterward, it was flushed with CO_2_ for 30 min. After electrochemical carbon capture, the cell was flushed again with N_2_ for at least 2 h to ensure a CO_2_-free system. Using cyclic voltammetry (CV) for carbon capture, the system was cycled in the reductive range between −1.05 and 0.15 V for 5 cycles at 50 mV s^–1^ after purging with N_2_ and CO_2_. After the second purging with N_2_, the CO_2_ was released by cycling in the oxidative range between 0.15 and 1.15 V for 5 cycles at a scan rate of 50 mV s^–1^. For chronoamperometric CO_2_ capture, after purging with CO_2_, the potential was kept constant at −0.85 V for 15 min. After the second purging of the cell with N_2_, the CO_2_ was released by applying a constant potential of 1.15 V for 15 min.

The Faradaic efficiency (FE) was calculated according to the following eq 1.

$$\mathrm{FE}=\frac{n\;z\;F}{Q}$$ 1

With n being the amount of CO_2_ detected during the release, z the number of electrons transferred (for carbon capture, z = 1), F the Faradaic constant and Q the total charge injected during the capture process.

The EIS measurements were carried out in a glass cell using a Biologic VMP3 potentiostat with a Pt CE and a Ag/AgCl reference electrode (0.197 V vs SHE). The 0.1 M phosphate buffer (pH 7 and 11) was purged with Ar or CO_2_ for at least 50 min before the electrochemical measurements. Prior to the EIS measurements, a CV was measured with a scan rate of 50 mV s^–1^ (5 cycles). EIS was carried out in a frequency range from 100 kHz to 10 mHz with a sinus amplitude of ± 10 mV and in equal potential steps of 100 mV from 0.8 to −1.0 V vs Ag/AgCl. The potential was maintained at a constant value for 30 min before the respective EIS measurements to achieve steady-state conditions. Fitting was done *via* EC-lab Software V 11.21 using a *Randomize+Simplex* approach with a weighed *Z.*

### 2.3 Characterization Methods

The amount of CO_2_ released during the carbon capture experiments was determined spectroscopically using a Bruker VERTEX 80-ATR infrared spectrometer. The scan was performed using a liquid nitrogen-cooled detector over a spectral range of 4000–500 cm^–1^, averaging 32 scans at a resolution of 2 cm^–1^ and an aperture setting of 1 mm. A gastight transmission cell with ZnSe windows was used to measure IR absorption in transmission mode. Prior to any measurement, the sample chamber was flushed with N_2_ to ensure a CO_2_-free environment. A sample from the headspace of the electrochemical cell was taken and analyzed in the transmission cell. After examining the CO_2_ content of the first sample in triplicate, an additional sample was taken from the cell and analyzed in triplicate as well. To quantify the amount of CO_2_ in the samples, calibration was performed using a defined amount of test gas (Linde, 5% CO_2_ in N_2_). The resulting IR spectra and calibration curve with the amount of substance present in the transmission cell can be found in Figure 1.

**Figure 1:**
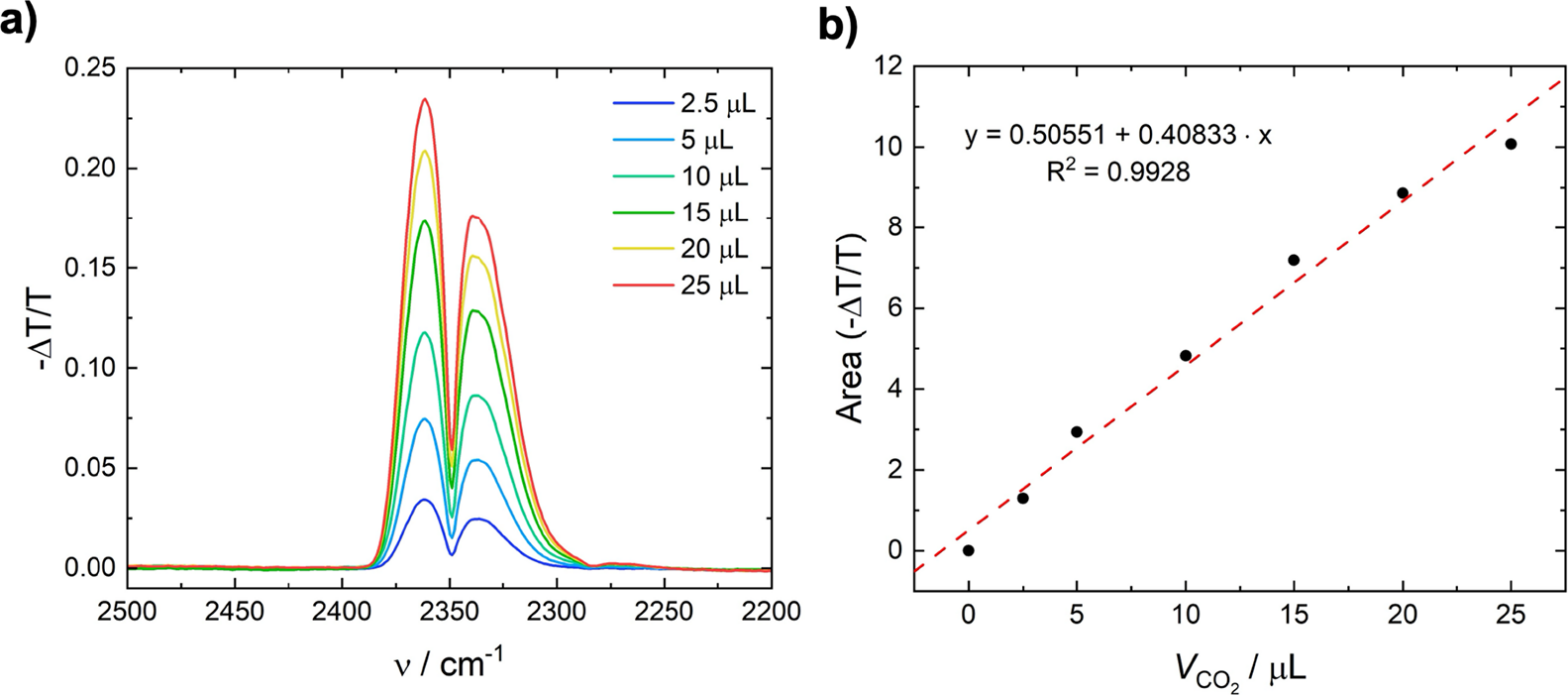
Calibration of the spectroscopic detection of CO_2_ using an FTIR and test gas with (a) spectra of different concentrations of CO_2_ and (b) calibration curve.

SEM images were obtained using a JEOL JSM-6360LV scanning electron microscope, operated under a high vacuum at an acceleration voltage of 7.0 kV. TGA was measured using a PerkinElmer TGA 4000 Thermogravimetric Analyzer at a heating rate of 10 °C min^–1^.

## 3 Results and Discussion

The CV of the polymerization of 1-AAQ on glassy carbon was recently published^46^ and can be found in Figure 2.

**Figure 2:**
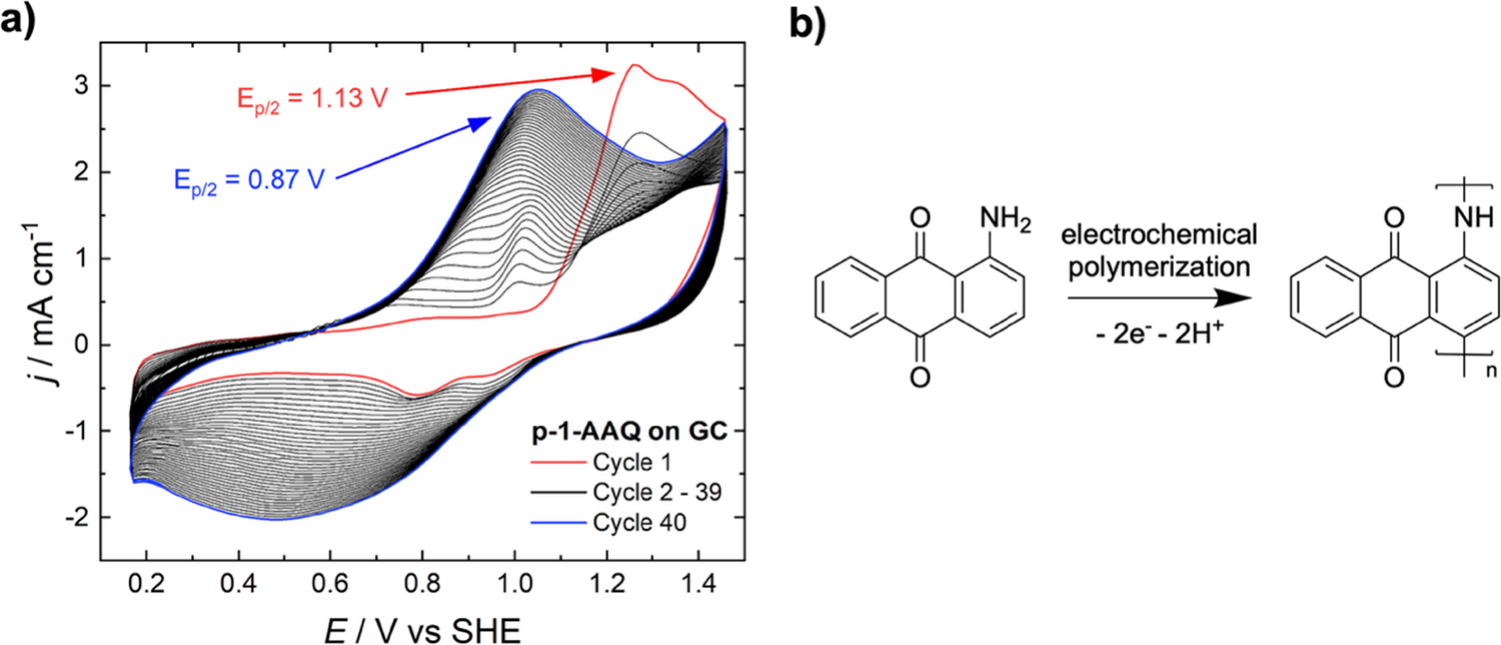
(a) CV of the polymerization of 1-AAQ on a glassy carbon electrode in 6 M H_2_SO_4_ and (b) polymerization reaction scheme.

The first cycle features an anodic peak at a half-step potential of 1.13 V, which is well-known from amino-substituted quinones.^48^ This peak is attributed to the first formation of the amino group to a radical cation.^45,49–51^ This cation starts the polymeric chain, to which further molecules are added by cycling the potential. The growth of the polymer can be monitored through the oxidative peak arising at 0.87 V. The resulting polymer was subsequently analyzed, and the ATR-FTIR spectrum is presented in Figure S1. The characteristic bands of the primary amine stretch, which are found in the monomeric form, change to the very broad band of the secondary amine in the polymeric spectrum. The complete assignment of the IR bands in both the monomeric and polymeric spectra can be found in Table S1. SEM images of the polymer are depicted in Figure 3. P-1-AAQ has a fluffy, sponge-like appearance on both glassy carbon and carbon paper electrodes, which is similar in appearance to electropolymerized polyaniline.^52^ Furthermore, sizable areas that remain uncoated by the polymer can be seen on both glassy carbon and carbon paper, to a similar degree. Detailed characterization of p-1-AAQ has been published recently.^46^

**Figure 3:**
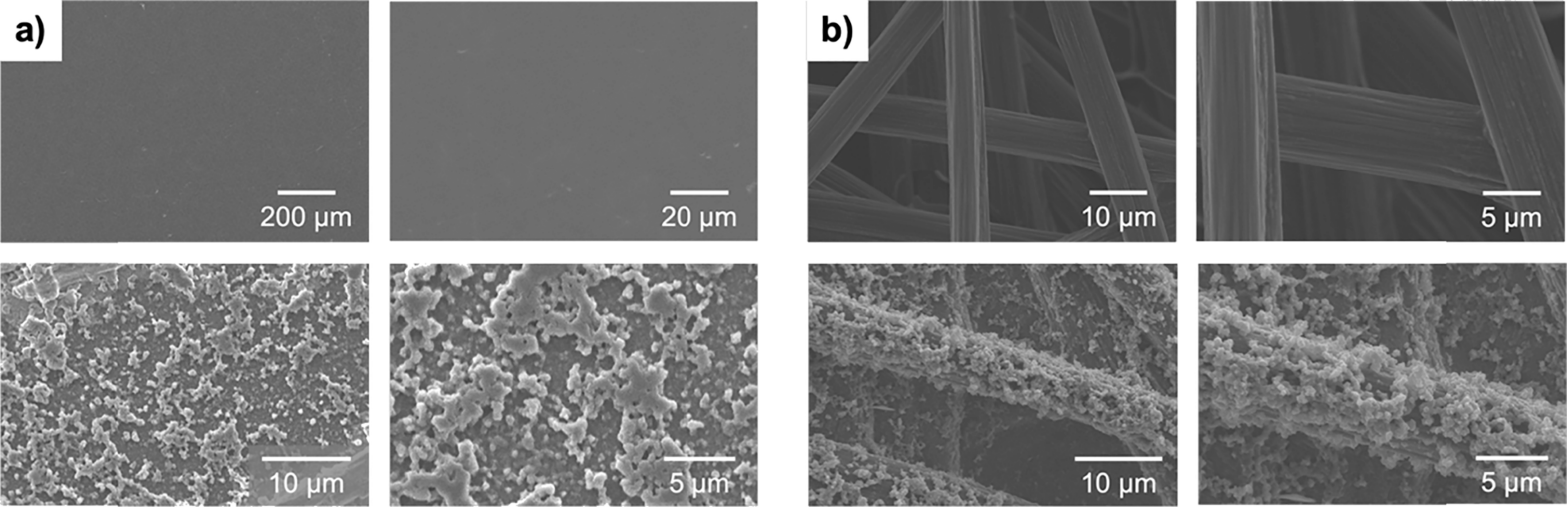
SEM images of blank (top) and polymer-coated (bottom) electrodes with (a) glassy carbon and (b) carbon paper electrodes.

Cyclic voltammograms were recorded under aqueous conditions to determine the suitability of p-1-AAQ for carbon capture. The CV of p-1-AAQ on both glassy carbon and carbon paper at an initial pH of 11 can be found in Figure 4. Under N_2_ saturated conditions, the CV of p-1-AAQ on a glassy carbon electrode shows a reversible peak at a half-step potential of −0.58 V. This reversible cathodic peak is observed similarly in aqueous solutions of monomeric, substituted anthraquinones and corresponds to a two-electron reduction which appears as one reductive peak in protic solvents.^53^ Upon adding CO_2,_ this peak decreases in current density and shifts toward more anodic potentials. Even though this shift can primarily be attributed to the change in pH upon the addition of CO_2_ as the pH value shifts from 11.0 to 9.2, it is assumed that it can also partly be attributed to the thus occurring capture of the CO_2_ molecule by the quinone group of the anthraquinone, as reported for nonaqueous solutions.^37,39,41^ Upon removing unbound CO_2_ from the solution by purging with N_2_ and a subsequent pH shift to 10.5, the half-step potential reverts to a more cathodic potential of −0.48 V. However, the current density does not recover and stays at a lower value compared to the initial CV under N_2_, which is in accordance with literature results.^34,54^

**Figure 4:**
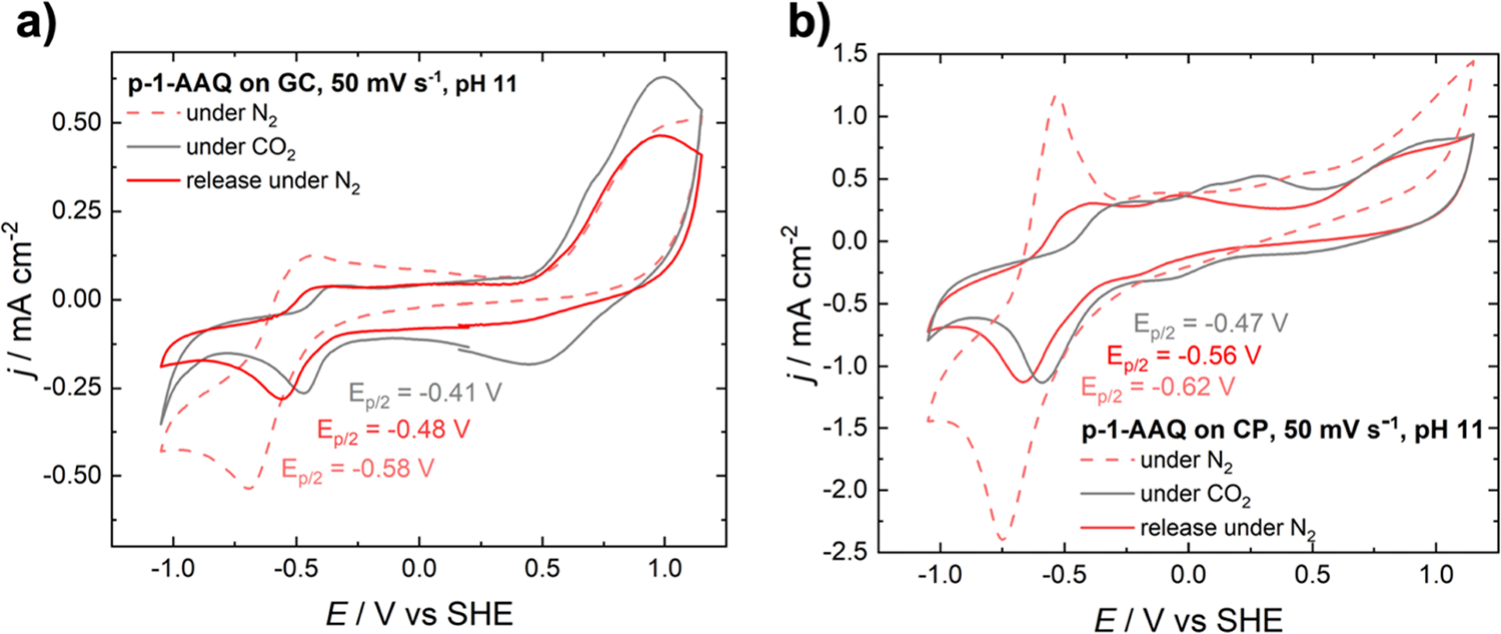
CVs of p-1-AAQ-coated electrodes in a phosphate buffer solution at pH 11 at a scan rate of 50 mV s^–1^ using a (a) glassy carbon and (b) carbon paper electrode.

Similar features can be observed when looking at the polymer-coated carbon paper electrode. The reductive peak, with a half-step potential of −0.62 V, reverts to a more anodic potential upon the introduction of CO_2_. Here, the decrease in current density can be observed quite prominently, with a decrease to approximately half the original value. Upon the subsequent removal of CO_2_ from the system, the reductive peak reverts to a half-step potential of −0.56 V. However, as with the p-1-AAQ-coated glassy carbon electrode, the current density does not increase again. A similar trend, with decreasing current densities and slight shifts in half-step potentials, can also be observed with carbon paper under neutral and acidic conditions, as depicted in Figure S2. Virtually no difference is observed in the CVs of glassy carbon at pH 2 and 7, as well as in the blank measurements at pH 7. For blank measurements, the electrodes were pretreated by cycling the potential using the same parameters as during the polymerization in 6 M sulfuric acid without any monomer present.

To further study the capability of p-1-AAQ toward carbon capture, quantitative analysis of the amount of CO_2_ captured and released has been performed. Two different methods for electrochemical capture and release have been employed. CV was first used to reductively capture and oxidatively release CO_2_. In the second method, a constant cathodic potential was employed to capture CO_2_, and a constant anodic potential was used to subsequently release it. This second method was named “electroswing”, according to the terminology used in literature.^42^ Results for the amount of CO_2_ captured and released and faradaic efficiencies of the carbon capture using the “electroswing” method can be found in Figure 5, with an example for the time versus current curves at a constant potential of −0.85 V.

**Figure 5:**
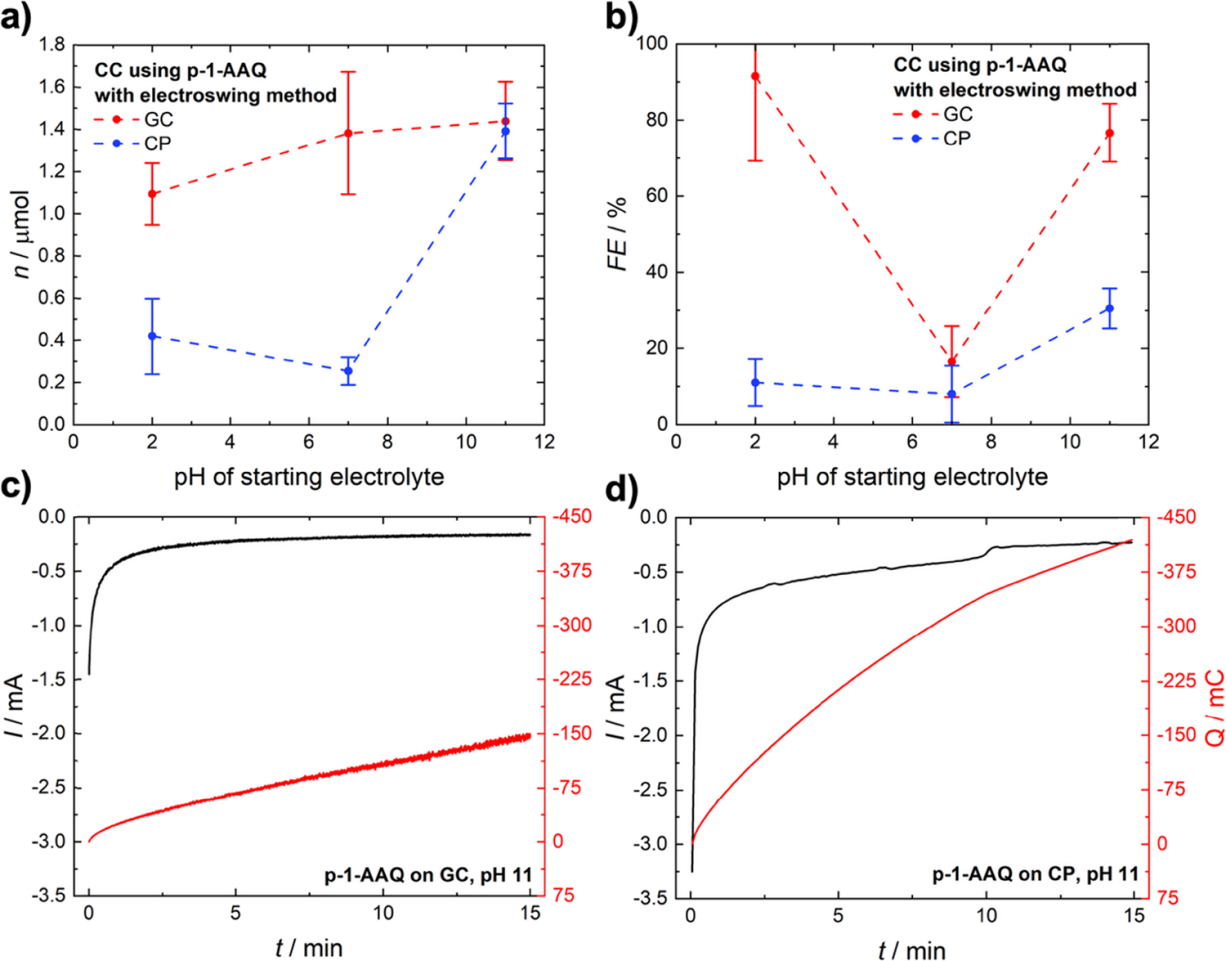
Results for electrochemical carbon capture (CC) using the “electroswing” method with (a) the amount of CO_2_ captured and released and (b) Faradaic efficiencies of the capture process at acidic, neutral, and alkaline pH levels and example of time vs current curves of the chronoamperometric measurements at −0.85 V for carbon capture using p-1-AAQ on a (c) glassy carbon and (d) carbon paper electrode in an alkaline buffer solution.

The results for the capture using CV can be found in Figure 6 together with an example of the CV method for carbon capture at alkaline conditions. Interestingly, employing glassy carbon electrodes yields a higher amount of CO_2_ captured and released as compared to carbon paper. This is true for both methods and all conditions. Most systems showed improved performance in highly acidic and alkaline conditions, while both the amount of CO_2_ captured and the efficiency of the capture process decreased under neutral conditions, with the only exception being a glassy carbon electrode used with the “electroswing” method. Here, the amount of CO_2_ captured steadily increases as the pH value rises. However, when looking at the Faradaic efficiency, it drastically increases under extreme conditions, with 92% at pH 2 and 77% at pH 11. This impressive efficiency under acidic conditions might be attributed to the increased intrinsic electrical conductivity of polyanthraquinone in higher acidic media. This behavior has been reported for polyaniline, which shares the same backbone as p-1-AAQ.^55–57^ A summary of the amount of CO_2_ captured and the faradaic efficiency of the capture can be found in Table S2. Additionally, blank measurements with pretreated electrodes were performed. Herein, faradaic efficiencies were below 8%, which can be seen as proof that the redox behavior of p-1-AAQ is essential for reversible CO_2_ capture and release process. Furthermore, to exclude other possible causes for the presence of CO_2_ after the release process, blank measurements were preformed including the polymer yet excluding any addition of CO_2_. As expected only minimal traces of CO_2_ were detected, being below the limit of quantification. Even though care was taken to exclude any atmospheric contamination, it is assumed that these traces of CO_2_ stem from the environment. CVs of the blank experiments as well as exact values can be found in Figures S3 and S4 as well as Tables S3 and S4.

**Figure 6:**
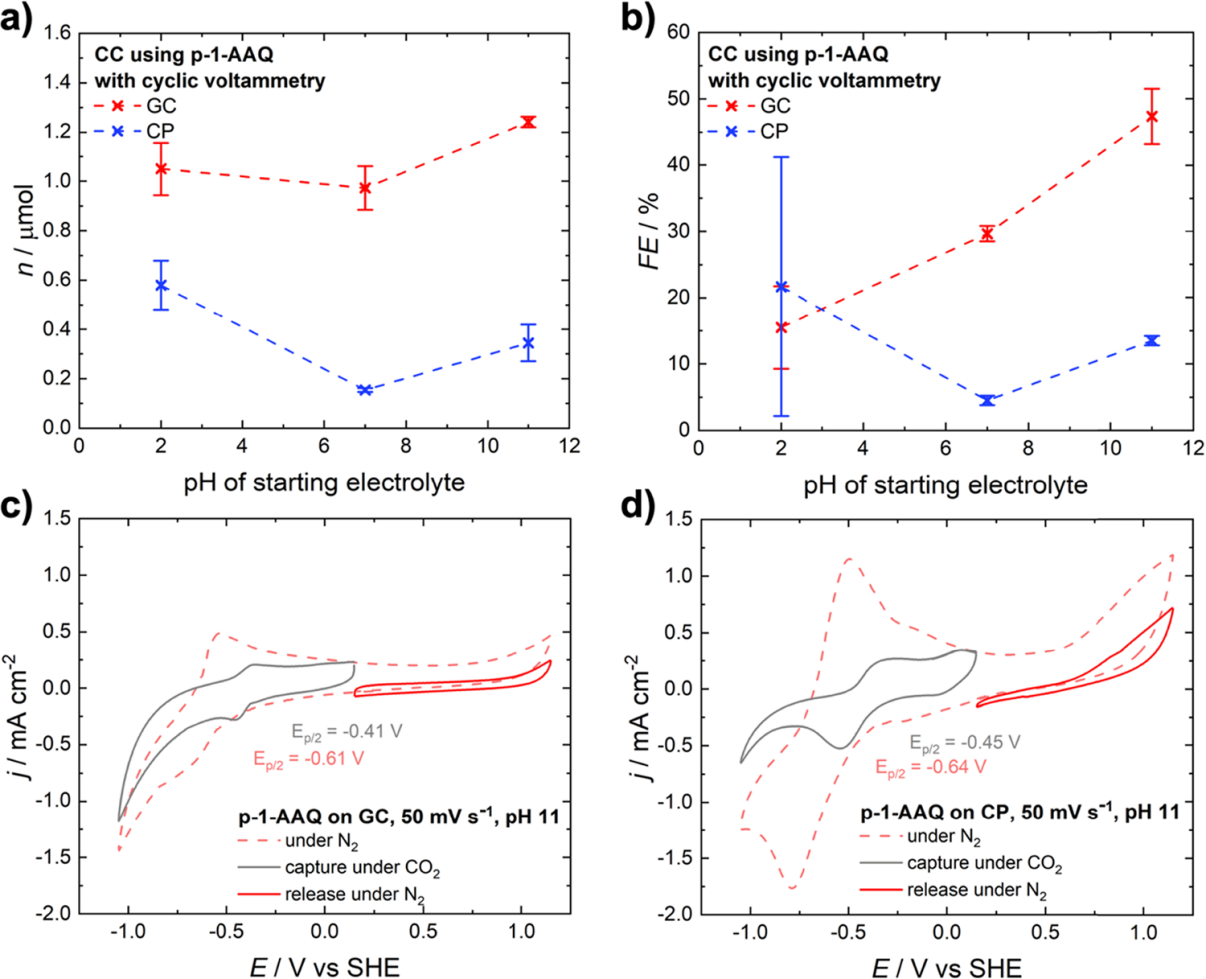
Quantitative evaluation of carbon capture with p-1-AAQ using cyclic voltammetry with (a) amount of CO_2_ captured and released and (b) faraday efficiency of carbon capture and capture CVs in alkaline electrolyte solution with (c) glassy carbon and (d) carbon paper.

TGA measurements of p-1-AAQ on top of carbon paper have been performed to estimate the amount of polymer present on the electrodes. The resulting thermogram can be found in Figure S5. Unfortunately, no clear, stepwise decrease in weight can be seen. However, it is assumed that all additional loss in weight as compared to the reference measurement can be attributed to the organic material on top of the electrode. Using a relative weight loss of 2.45% as the deposited amount of p-1-AAQ on carbon paper, calculations of CO_2_-uptake capacities per polymer repeating unit were performed. Thereby we found that an uptake of 2.85 mmol_CO2_ g_p‑1‑AAQ_^–1^ was possible for the CV method and 6.85 mmol_CO2_ g_p‑1‑AAQ_^–1^ for the “electroswing” method when using a carbon paper electrode. These values are comparable to the adsorption capacities reported in our previous study that employ evaporated anthraquinone thin films for carbon capture,^34^ while the value found when using the “electroswing” method even exceeded the previous CO_2_ uptake capacities. A study showed that 2-aminoanthraquinone covalently immobilized onto a porous carbon support was able to capture 4.44 mmol_CO2_ g_AQ_^–1^, which was further improved in a subsequent study.^35^ Herein, significantly higher quinone loadings up to 10 wt % were achieved, resulting in a reported molar adsorption capacity of >60%, displaying the anthraquinone’s ability to capture 2 CO_2_ molecules per mol anthraquinone.^36^ Similar values have been reported for another polyanthraquinone-carbon nanotube composite, with an uptake efficiency of 65%^42^ and for anthraquinone thin films, with an uptake efficiency of 61%.^34^

When using a p-1-AAQ carbon paper electrode with the CV method described in this present work, a slightly lower uptake efficiency of 32% is found. However, when employing the “electroswing” method, an astounding uptake efficiency of 76% is observed. This value is among the highest reported up to now and in the upcoming paragraphs we tried to further understand this exemplary behavior of p-1-AAQ. Thus, our results demonstrate the electrochemical addressability of the polymer on top of the electrode surface as well as underscore the capability of p-1-AAQ to capture 2 CO_2_ molecules per repeating unit under certain conditions.

P-1-AAQ not only excels in molar uptake efficiency but also achieves gravimetric CO_2_ capacities of 0.17 mmol_CO2_ g_p‑1‑AAQ+CP_^–1^. These values approach those observed in covalently functionalized carbon materials.^35,36^ Notably, while prior studies typically involve up to 10 wt % quinone loading, p-1-AAQ contains only about 2.5 wt % of the total electrode mass, indicating the significant potential for further enhancement.

The observation that the amount of CO_2_ captured between glassy carbon and carbon paper at pH 11 (Figure 5a) is nearly identical, while at the same time, the Faradaic efficiency at the glassy carbon-based electrode is significantly higher than that of carbon paper (Figure 5b) when using the “electroswing” method, is intriguing and not yet fully understood. To gain further insights into this phenomenon, we conducted potential-dependent electrochemical impedance spectroscopy (PEIS) on the p-1-AAQ-coated electrodes under Ar and CO_2_-saturated electrolytes at pH 11 (Figure 7).

**Figure 7:**
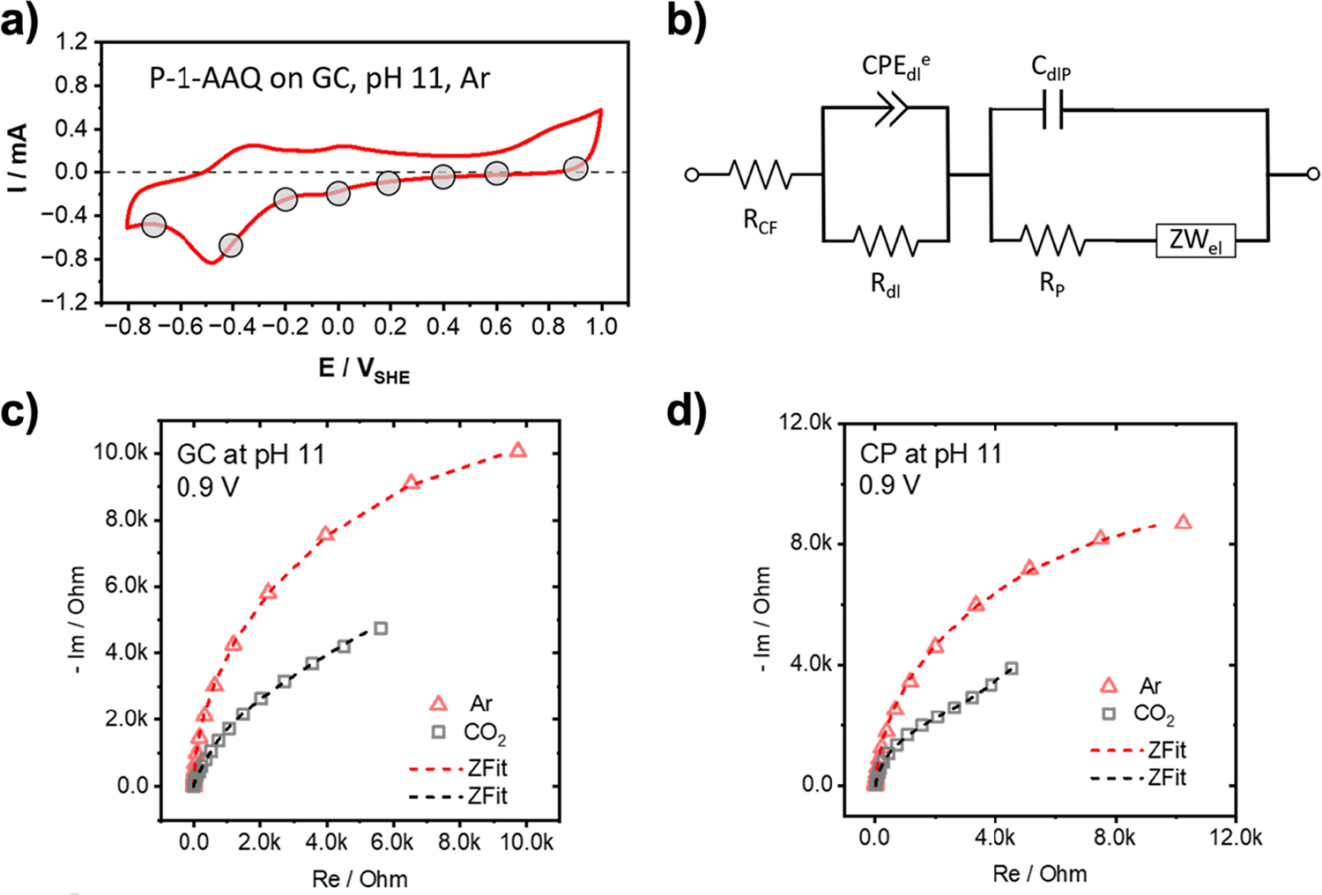
Potential-dependent electrochemical impedance spectroscopy: (a) CVs of p-1-AAQ-coated electrodes in a phosphate buffer solution at pH 11 at a scan rate of 50 mV s^–1^ using a glassy carbon electrode under Ar saturation, with gray circles indicating the potentials of EIS data acquisition and impedance fit analysis. (b) Equivalent electric circuit used for fitting the EIS data. (c) and (d), example Nyquist plots at 0.7 V for p-1-AAQ-coated glassy carbon and carbon paper electrodes, respectively. Symbols represent the experimental data, and the lines are the best fit.

For additional quantification of the measured EIS data, corresponding electronic elements were determined by fitting the experimental spectra of selected potentials (Figure 7 **a,** gray circles) to the proposed equivalent circuit depicted in Figure 7b. In Figure 7c and **d**, example Nyquist plots at 0.7 V for the measured and fitted p-1-AAQ-coated glassy carbon and carbon paper electrodes, under Ar and CO_2_ saturated electrolyte solutions, are shown, respectively. A detailed summary of all fitting parameters and their corresponding mean square deviations is given in Tables S6–S9 in the Supporting Information.

Care was taken to ensure that all parameters used for fitting the data have a well-defined physical meaning, ensuring the model’s consistency with the underlying physical principles. The initial equivalent circuit (Figure S6) proposed two interfaces in parallel that contribute to the electrochemical response, namely one between the carbon that is not covered with p-1-AAQ and the electrolyte and the second between the p-1-AAQ and the electrolyte. Interestingly, this initial idea was not able to fit the recorded EIS spectra satisfactorily, as parameters take on physically unrealistic values while still resulting in high weighted residuals (for example, the resistance reaches ∼ 3.776 × 10^27^ Ω with a weighted residual of Z = 0.049, Figure S7b) or the fit yields physically impossible parameter values (for example, a negative capacitance, Figure S7a). A modification, putting the two interfaces in serial connection (Figure 7b), has proven to successfully represent the experimental measurements. A comparison of both models can be found in Figure S8 and Table S9. The successful implementation of this second model suggests that the electrochemical reactions between uncovered carbon fibers and the electrolyte are likely minor, and that the EC signal is predominantly influenced by interactions between p-1-AAQ and the electrolyte. Results for the fitted data can be found in Figure 8.

**Figure 8:**
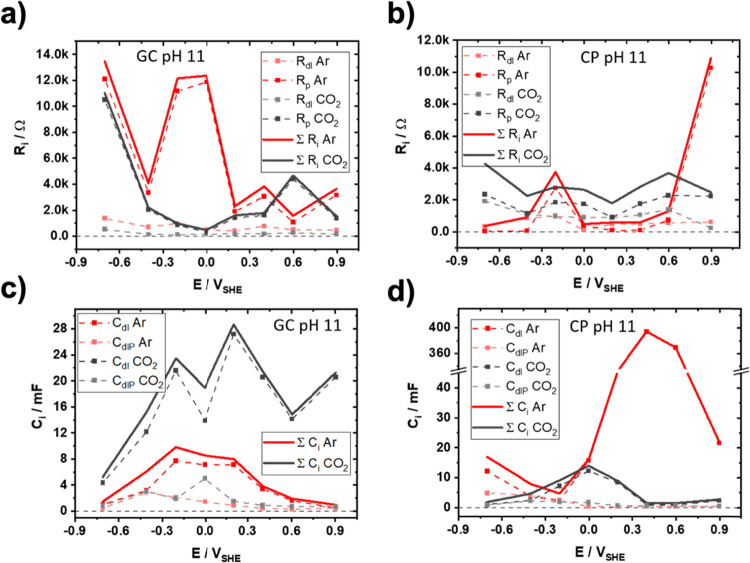
Potential-dependent electrochemical impedance spectroscopy: (a, b) potential-dependent electrical parameters of the Ohmic carbon electrode/electrolyte interface resistance (*R*_dl_), as well as the interface resistance between p-1-AAQ and the electrolyte (*R*_p_) and (c, d) potential dependent electrical parameters of the double-layer capacitance (*C*_dl_) and interface capacitance (*C*_dhP_), for p-1-AAQ-coated glassy carbon and carbon paper electrodes, respectively.

From the EIS data, we concluded that the ohmic resistance of the electrolyte solution and carbon current collector (R_CF_) is almost constant at all potentials, pH values, and Ar or CO_2_ saturation conditions, fluctuating only slightly (between 3.5 and 12.7 Ω, Table S5–S8). The interfacial carbon electrode/p-1-AAQ electron charge transfer resistance (R_dl_) remains relatively constant across all measurements and all potential ranges, as expected from the solid/solid interface between the carbon current collector and the p-1-AAQ polymer. The overall potential dependent resistance (ΣR_i_ Ar and R_i_ CO_2_, Figure 8a and **b**) is dominated by the interface resistance of the p-1-AAQ polymer and the electrolyte (R_p_).

The amount of CO_2_ captured between glassy carbon and carbon paper at pH 11 (Figure 5a) is almost equal. At the same time, the Faradaic efficiency at the glassy carbon-based electrode is significantly higher than that of carbon paper using the “electroswing” method. This can be readily understood from the potential dependent impedance data. At the CO_2_ capture potential (−0.4 to −0.6 V), the interface capacitance between p-1-AAQ and the CO_2_ saturated electrolyte (C_dlP_ CO_2_) is quite similar between glassy carbon and carbon paper with ≈ 2 – 3 mF, Figure 8c and **d**, so is the corresponding R_p_ (≈ 2 kΩ), indicating a similar characteristic for the CO_2_ captured. However, at lower potentials (−0.9 V), used for the “electroswing” experiment (Figure 5), the R_p_ of glassy carbon increases significantly to 10.5 kΩ, while it remains almost constant for the carbon paper electrode at 2.3 kΩ (Figure 8a and **b**). This lower R_p_ resistance at −0.9 V allows for more current at that potential, which is related to unwanted side reactions, i.e., H_2_ evolution, as corroborated by the higher current and charge accumulated in the chronoamperometric capture experiment with 150 vs 400 mC between glassy carbon and carbon paper-based electrodes, respectively (Figure 5).

For the PEIS measurements of p-1-AAQ on glassy carbon at pH 11 and under CO_2_ saturation, an additional impedance signal at high frequencies, between 30.7 kHz and 4.3 kHz, is measured, represented by an additional semicircle in the corresponding Nyquist plot (Figures S6). This high-frequency impedance response remains constant over all measured potentials and can be represented with an ohmic resistance of 3.04 Ω in parallel to a capacitance with 1.2 μF (X^2^/|Z| of 3.8 × 10^–3^). Since there is no change upon the applied potential and the frequency range is very high, it can be attributed to an electronic contact interface unrelated to the CO_2_ capture capabilities of p-1-AAQ on glassy carbon.

Although very frequently applied in battery research, impedance analysis for electrochemical CO_2_ capture and release is a relatively rarely used technique. A literature review revealed few if any, comparable studies—apart from a previous investigation by Apaydin et al.^58^ conducted within our research group. In that study, an organic semiconductor electrode was introduced for the electrochemical capture of dissolved CO_2_ in aqueous electrolytes. The process is based on the electrochemical reduction of a thin film composed of a naphthalene bisimide derivative, specifically 2,7-bis­(4-(2-(2-ethylhexyl)­thiazol-4-yl)­phenyl)­benzo­[lmn]­[3,8]­phenanthroline-1,3,6,8­(2H,7H)-tetraone (NBIT).

PEIS was also performed in that study to further investigate the redox behavior of the NBIT thin films. Interestingly, in that system, EIS revealed a decrease in the overall charge transfer resistance (R_ct_) with increasing negative potential under both Ar and CO_2_ atmospheres. This observation contrasts with our findings for the p-1-AAQ system (Figure 8a and **b**).

This discrepancy can be attributed to a) the different nature of the active materials used and b) the different electrolytes employed in the previous study, which consisted of a 0.1 M Na_2_SO_4_ solution without any buffering agent. This results in a significantly different pH (approximately pH 5), which is expected to shift further toward acidic values under CO_2_-saturated conditions.

## 4 Conclusions

This study demonstrates the high potential of p-1-AAQ as an effective material for carbon capture. The polymer is made from inexpensive raw materials through a simple oxidative electrochemical polymerization process. The CV and “electroswing” methods revealed that the glassy carbon electrode exhibits higher Faradaic efficiency than carbon paper despite similar CO_2_ capture amounts when using the “electroswing” method at pH 11. PEIS provided insights into the electrode/polymer interface, indicating that the interface resistance between the polymer and the electrolyte plays a critical role in CO_2_ capture efficiency. While both electrodes show comparable interface capacitance at moderate potentials, the significantly higher R_p_ at lower potentials for glassy carbon reduces unwanted side reactions such as hydrogen evolution.

The “electroswing” method outperforms the CV method in terms of CO_2_ capture capacity, particularly under acidic and alkaline conditions, with a maximum uptake efficiency of 76 %. Herein, we demonstrate the capture of 2 CO_2_ molecules per polymeric repeating unit. P-1-AAQ not only outperforms previous material classes in terms of molar uptake efficiency but also displays gravimetric CO_2_ capacities of 0.17 mmol_CO2_ g_p‑1‑AAQ+CP_^–1^ which are close to covalently functionalized carbons. However, while literature reports up to 10 wt % loading of quinones for their results, p-1-AAQ was determined to only bear roughly 2.5 wt % of the total electrode mass, which leaves room for further optimization. The improved performance at extreme pH values can be attributed to the enhanced conductivity and protonation states of the p-1-AAQ polymer. The study highlights that optimizing electrode materials and operating conditions is crucial for maximizing CO_2_ capture efficiency. Additionally, we identified an interesting behavior of the polymer, where the charge transfer between the uncovered areas of the electrode and the electrolyte solution is negligible compared to the charge transfer between the polymer and the electrolyte solution. Furthermore, ionic or electronic diffusion within the polymer seems to be insignificant compared to diffusion within the electrolyte solution. This finding is also supported by the comparably high uptake efficiency, showing that the polymeric coating is readily electrochemically addressable through the carbon electrodes. We hope that these results encourage the scientific community to also implement PEIS studies as a regular characterization type for future electrochemical CO_2_ capture work.

Future work should focus on improving the uniformity of the polymer coating to minimize uncoated electrode areas, thereby further enhancing CO_2_ capture performance. Additionally, understanding the role of electrode surface morphology in impedance characteristics will aid in developing more efficient carbon capture systems toward large-scale application.

## Supplementary Material



## Notes

Figure S1: IR spectra and image of p-1-AAQ on FTO; Table S1: peak assignment of IR spectra; Figure S2: CVs of p-1-AAQ; Table S2: capture efficiency of p-1-AAQ; Figure S3: CVs of pretreated electrodes; Table S3: capture efficiency of pretreated electrodes; Figure S4: CVs without CO_2_; Table S4: capture efficiency without CO_2_; Figure S5: TGA; Tables S5–S8: fitting parameters for PEIS; Figure S6: schematic drawing of equivalent circuit; Figures S7 and S8 and Table S9: comparison of equivalent circuits; Figure S9: high-frequency impedance signal (PDF)

## References

[1] Abbass K., Qasim M. Z., Song H., Murshed M., Mahmood H., Younis I. (2022). A Review of the Global Climate Change Impacts, Adaptation,
and Sustainable Mitigation Measures. Environ.
Sci. Pollut Res..

[2] Friedlingstein P., Jones M. W., O’Sullivan M., Andrew R. M., Bakker D. C. E., Hauck J., Le Quéré C., Peters G. P., Peters W., Pongratz J., Sitch S., Canadell J. G., Ciais P., Jackson R. B., Alin S. R., Anthoni P., Bates N. R., Becker M., Bellouin N., Bopp L., Chau T. T. T., Chevallier F., Chini L. P., Cronin M., Currie K. I., Decharme B., Djeutchouang L. M., Dou X., Evans W., Feely R. A., Feng L., Gasser T., Gilfillan D., Gkritzalis T., Grassi G., Gregor L., Gruber N., Gürses Ö., Harris I., Houghton R. A., Hurtt G. C., Iida Y., Ilyina T., Luijkx I. T., Jain A., Jones S. D., Kato E., Kennedy D., Goldewijk K. K., Knauer J., Korsbakken J. I., Körtzinger A., Landschützer P., Lauvset S. K., Lefèvre N., Lienert S., Liu J., Marland G., McGuire P. C., Melton J. R., Munro D. R., Nabel J. E. M. S., Nakaoka S. I., Niwa Y., Ono T., Pierrot D., Poulter B., Rehder G., Resplandy L., Robertson E., Rödenbeck C., Rosan T. M., Schwinger J., Schwingshackl C., Séférian R., Sutton A. J., Sweeney C., Tanhua T., Tans P. P., Tian H., Tilbrook B., Tubiello F., Van Der Werf G. R., Vuichard N., Wada C., Wanninkhof R., Watson A. J., Willis D., Wiltshire A. J., Yuan W., Yue C., Yue X., Zaehle S., Zeng J. (2022). Global Carbon Budget 2021. Earth Syst. Sci.
Data.

[3] Holechek J. L., Geli H. M. E., Sawalhah M. N., Valdez R. (2022). A Global Assessment:
Can Renewable Energy Replace Fossil Fuels by 2050?. Sustainability.

[4] Tol R. S. J. (2018). The
Economic Impacts of Climate Change. Rev. Environ.
Econ. Policy.

[5] Zhao Y., Liu S. (2023). Effects of Climate Change on Economic Growth: A Perspective of the
Heterogeneous Climate Regions in Africa. Sustainability.

[6] Gao W., Liang S., Wang R., Jiang Q., Zhang Y., Zheng Q., Xie B., Toe C. Y., Zhu X., Wang J., Huang L., Gao Y., Wang Z., Jo C., Wang Q., Wang L., Liu Y., Louis B., Scott J., Roger A. C., Amal R., He H., Park S. E. (2020). Industrial Carbon Dioxide Capture and Utilization:
State of the Art and Future Challenges. Chem.
Soc. Rev..

[7] Challa P., Paleti G., Madduluri V. R., Gadamani S. B., Pothu R., Burri D. R., Boddula R., Perugopu V., Kamaraju S. R. R. (2022). Trends
in Emission and Utilization of CO_2_: Sustainable Feedstock
in the Synthesis of Value-Added Fine Chemicals. Catal. Surv. Asia.

[8] Rochelle G. T. (2009). Amine Scrubbing
for CO_2_ Capture. Science.

[9] Barzagli F., Mani F., Peruzzini M. (2016). A Comparative
Study of the CO_2_ Absorption in Some Solvent-Free Alkanolamines
and in Aqueous
Monoethanolamine (MEA). Environ. Sci. Technol..

[10] Wadi B., Golmakani A., Manovic V., Nabavi S. A. (2021). Effect of Combined
Primary and Secondary Amine Loadings on the Adsorption Mechanism of
CO_2_ and CH_4_ in Biogas. Chem. Eng. J..

[11] Meng F., Meng Y., Ju T., Han S., Lin L., Jiang J. (2022). Research Progress of Aqueous Amine Solution for CO_2_ Capture:
A Review. Renew. Sustain. Energy Rev..

[12] Choi G. H., Song H. J., Lee S., Kim J. Y., Moon M. W., Yoo P. J. (2023). Electrochemical
Direct CO_2_ Capture Technology
Using Redox-Active Organic Molecules to Achieve Carbon-Neutrality. Nano Energy.

[13] Diederichsen K. M., Sharifian R., Kang J. S., Liu Y., Kim S., Gallant B. M., Vermaas D., Hatton T. A. (2022). Electrochemical
Methods for Carbon Dioxide Separations. Nat.
Rev. Methods Primers.

[14] Li X., Zhao X., Liu Y., Hatton T. A., Liu Y. (2022). Redox-Tunable
Lewis Bases for Electrochemical Carbon Dioxide Capture. Nat. Energy.

[15] Rosen N., Welter A., Schwankl M., Plumeré N., Staudt J., Burger J. (2024). Assessment of the Potential of Electrochemical
Steps in Direct Air Capture through Techno-Economic Analysis. Energy Fuels.

[16] Huebscher R. G., Babinsky A. D. (1969). Electrochemical
Concentration and Separation of Carbon
Dioxide For Advanced Life Support Systems-Carbonation Cell System. SAE Trans..

[17] Zito A. M., Clarke L. E., Barlow J. M., Bím D., Zhang Z., Ripley K. M., Li C. J., Kummeth A., Leonard M. E., Alexandrova A. N., Brushett F. R., Yang J. Y. (2023). Electrochemical
Carbon Dioxide Capture and Concentration. Chem.
Rev..

[18] Appel A. M., Newell R., DuBois D. L., Rakowski
DuBois M. (2005). Concentration
of Carbon Dioxide by Electrochemically Modulated Complexation with
a Binuclear Copper Complex. Inorg. Chem..

[19] Renfrew S. E., Starr D. E., Strasser P. (2020). Electrochemical
Approaches toward
CO_2_ Capture and Concentration. ACS
Catal..

[20] Scovazzo P., Poshusta J., DuBois D., Koval C., Noble R. (2003). Electrochemical
Separation and Concentration of 1% Carbon Dioxide from Nitrogen. J. Electrochem. Soc..

[21] Gurkan B., Simeon F., Hatton T. A. (2015). Quinone
Reduction in Ionic Liquids
for Electrochemical CO_2_ Separation. ACS Sustain. Chem. Eng..

[22] Rheinhardt J. H., Singh P., Tarakeshwar P., Buttry D. A. (2017). Electrochemical
Capture and Release of Carbon Dioxide. ACS Energy
Lett..

[23] Comeau
Simpson T., Durand R. R. (1990). Reactivity of Carbon Dioxide with
Quinones. Electrochim. Acta.

[24] Gupta N., Linschitz H. (1997). Hydrogen-Bonding
and Protonation Effects in Electrochemistry
of Quinones in Aprotic Solvents. J. Am. Chem.
Soc..

[25] Diederichsen K. M., Liu Y., Ozbek N., Seo H., Hatton T. A. (2022). Toward Solvent-Free
Continuous-Flow Electrochemically Mediated Carbon Capture with High-Concentration
Liquid Quinone Chemistry. Joule.

[26] Werner D., Apaydin D. H., Portenkirchner E. (2018). An Anthraquinone/Carbon
Fiber Composite
as Cathode Material for Rechargeable Sodium-Ion Batteries. Batter. Supercaps.

[27] Friebe C., Lex-Balducci A., Schubert U. S. (2019). Sustainable Energy Storage: Recent
Trends and Developments toward Fully Organic Batteries. ChemSusChem.

[28] Werner D., Apaydin D. H., Wielend D., Geistlinger K., Saputri W. D., Griesser U. J., Drazevic E., Hofer T. S., Portenkirchner E. (2021). Analysis of the Ordering Effects in Anthraquinone Thin
Films and Its Potential Application for Sodium Ion Batteries. J. Phys. Chem. C.

[29] Li H., Dilipkumar A., Abubakar S., Zhao D. (2023). Covalent Organic Frameworks
for CO_2_ Capture: From Laboratory Curiosity to Industry
Implementation. Chem. Soc. Rev..

[30] Li X., Mathur A., Liu A., Liu Y. (2023). Electrifying Carbon
Capture by Developing Nanomaterials at the Interface of Molecular
and Process Engineering. Acc. Chem. Res..

[31] Le P. H., Liu A., Zasada L. B., Geary J., Kamin A. A., Rollins D. S., Nguyen H. A., Hill A. M., Liu Y., Xiao D. J. (2025). Nitrogen-Rich
Conjugated Macrocycles: Synthesis, Conductivity, and Application in
Electrochemical CO_2_ Capture. Angew.
Chem. Int. Ed..

[32] Rybak A., Rybak A., Boncel S., Kolanowska A., Kaszuwara W., Kolev S. D. (2022). Hybrid Organic-Inorganic Membranes
Based on Sulfonated Poly (Ether Ether Ketone) Matrix and Iron-Encapsulated
Carbon Nanotubes and Their Application in CO_2_ Separation. RSC Adv..

[33] Park Y. J., Lee H., Choi H. L., Tapia M. C., Chuah C. Y., Bae T. H. (2023). Mixed-Dimensional
Nanocomposites Based on 2D Materials for Hydrogen Storage and CO_2_ Capture. NPJ. 2D Mater. Appl..

[34] Wielend D., Apaydin D. H., Sariciftci N. S. (2018). Anthraquinone
Thin-Film Electrodes
for Reversible CO_2_ Capture and Release. J. Mater. Chem. A.

[35] Hartley N. A., Pugh S. M., Xu Z., Leong D. C. Y., Jaffe A., Forse A. C. (2023). Quinone-Functionalised Carbons as New Materials for
Electrochemical Carbon Dioxide Capture. J. Mater.
Chem. A.

[36] Hartley N.
A., Xu Z., Kress T., Forse A. C. (2024). Correlating the Structure of Quinone-Functionalized
Carbons with Electrochemical CO_2_ Capture Performance. Mater. Today Energy.

[37] Simeon F., Stern M. C., Diederichsen K. M., Liu Y., Herzog H. J., Hatton T. A. (2022). Electrochemical and Molecular Assessment
of Quinones
as CO_2_-Binding Redox Molecules for Carbon Capture. J. Phys. Chem. C.

[38] Bui A. T., Hartley N. A., Thom A. J. W., Forse A. C. (2022). Trade-Off between
Redox Potential and the Strength of Electrochemical CO_2_ Capture in Quinones. J. Phys. Chem. C.

[39] Schimanofsky C., Wielend D., Kröll S., Lerch S., Werner D., Gallmetzer J. M., Mayr F., Neugebauer H., Irimia-Vladu M., Portenkirchner E., Hofer T. S., Sariciftci N. S. (2022). Direct
Electrochemical CO_2_ Capture Using Substituted Anthraquinones
in Homogeneous Solutions: A Joint Experimental and Theoretical Study. J. Phys. Chem. C.

[40] Iida H., Kondou S., Tsuzuki S., Tashiro M., Shida N., Motokura K., Dokko K., Watanabe M., Ueno K. (2023). Imidazolium-Functionalized
Anthraquinone for High-Capacity Electrochemical CO_2_ Capture. J. Phys. Chem. C.

[41] Barlow J. M., Yang J. Y. (2022). Oxygen-Stable Electrochemical
CO_2_ Capture
and Concentration with Quinones Using Alcohol Additives. J. Am. Chem. Soc..

[42] Voskian S., Hatton T. A. (2019). Faradaic Electro-Swing
Reactive Adsorption for CO_2_ Capture. Energy Environ. Sci..

[43] Hemmatifar A., Kang J. S., Ozbek N., Tan K. J., Hatton T. A. (2022). Electrochemically
Mediated Direct CO_2_ Capture by a Stackable Bipolar Cell. ChemSusChem.

[44] Schubert, U. S. ; Winter, A. ; Newkome, G. R. An Introduction to Redox Polymers for Energy-Storage Applications; John Wiley & Sons: 2023.

[45] Badawy W. A., Ismail K. M., Medany S. S. (2006). Optimization of
the Electropolymerization
of 1-Amino-9,10-Anthraquinone Conducting Films from Aqueous Media. Electrochim. Acta.

[46] Kleinbruckner N., Leeb E., Wielend D., Schimanofsky C., Cobet M., Mayr F., Kerschbaumer A., Yumusak C., Richtar J., Scharber M. C., Neugebauer H., Irimia-Vladu M., Krajcovic J., Sariciftci N. S. (2024). Polymerized
Riboflavin and Anthraquinone Derivatives for Oxygen Reduction Reaction. Adv. Sustain. Syst..

[47] Wittstock, G. Lehrbuch Der Elektrochemie: Grundlagen, Methoden, Materialien, Anwendungen; John Wiley & Sons: 2023.

[48] Werner D., Greussing V., Pattis D., Gallmetzer J. M., Schimanofsky C., Wielend D., Liebl S., Stüwe T., Ciganek M., Krajčovič J., Irimia-Vladu M., Hofer T. S., Portenkirchner E. (2025). Towards the All Organic Na-Ion Battery,
Using Naturally Occurring Amino- and Hydroxy Substituted Anthraquinones. Electrochim. Acta.

[49] Wei Y., Sun Y., Tang X. (1989). Autoacceleration
and Kinetics of Electrochemical Polymerization
of Aniline. J. Phys. Chem..

[50] Ohsaka T., Ohba M., Sato M., Oyama N., Tanaka S., Nakamura S. (1991). Formation of a Novel
Electroactive Film by Electropolymerization
of 5-Amino-1-Naphthol. J. Electroanal. Chem..

[51] Gao M., Yang F., Wang X., Zhang G., Liu L. (2007). Electrochemical
Characteristics and Stability of Poly­(1,5-Diaminoanthraquinone) in
Acidic Aqueous Solution. J. Phys. Chem. C.

[52] Rabl H., Wielend D., Tekoglu S., Seelajaroen H., Neugebauer H., Heitzmann N., Apaydin D. H., Scharber M. C., Sariciftci N. S. (2020). Are Polyaniline
and Polypyrrole Electrocatalysts for
Oxygen (O_2_) Reduction to Hydrogen Peroxide (H_2_O_2_)?. ACS Appl. Energy Mater..

[53] Kerschbaumer A., Wielend D., Leeb E., Schimanofsky C., Kleinbruckner N., Neugebauer H., Irimia-Vladu M., Sariciftci N. S. (2023). How to Use a Rotating Ring-Disc Electrode (RRDE) Subtraction
Method to Investigate the Electrocatalytic Oxygen Reduction Reaction?. Catal. Sci. Technol..

[54] Apaydin D. H., Głowacki E. D., Portenkirchner E., Sariciftci N. S. (2014). Direct
Electrochemical Capture and Release of Carbon Dioxide Using an Industrial
Organic Pigment: Quinacridone. Angew. Chem.
Int. Ed..

[55] Osaka T., Nakajima T., Naoi K., Owens B. B. (1990). Electroactive Polyaniline
Film Deposited from Nonaqueous Organic Media II. Effect of Acid Concentration
in Solution. J. Electrochem. Soc..

[56] Blinova N.
V., Stejskal J., Trchová M., Prokeš J. (2008). Control of
Polyaniline Conductivity and Contact Angles by Partial Protonation. Polym. Int..

[57] Cao Y., Andreatta A., Heeger A. J., Smith P. (1989). Influence of Chemical
Polymerization Conditions on the Properties of Polyaniline. Polymer.

[58] Apaydin D. H., Gora M., Portenkirchner E., Oppelt K. T., Neugebauer H., Jakesova M., Głowacki E. D., Kunze-Liebhäuser J., Zagorska M., Mieczkowski J., Sariciftci N. S. (2017). Electrochemical
Capture and Release of CO_2_ in Aqueous Electrolytes Using
an Organic Semiconductor Electrode. ACS Appl.
Mater. Interfaces.

