# Supplementary material for: Reversible Carbon Dioxide Capture and Release using an Electropolymerized Anthraquinone Electrode in Aqueous Solution
Source: ACS Appl Mater Interfaces. 2025 Oct 8;17(42):58363–73. doi: 10.1021/acsami.5c17350 (PMC12557215; doi:10.1021/acsami.5c17350)
Supplement: Supplementary file 1 [file am5c17350_si_001.pdf]

Supplementary information

Reversible Carbon Dioxide Capture and Release using an Electropolymerized Anthraquinone Electrode in Aqueous Solution

Elisabeth Leeb[1,], Dominik Wielend[1], Nadine Kleinbruckner[1], Daniel Werner[2], Corina Schimanofsky[1], Victoria Greussing[2], Katharina Matura[1], Engelbert Portenkirchner[2], Niyazi Serdar Sariciftci[1]*

[1]Linz Institute for Organic Solar Cells (LIOS), Institute of Physical Chemistry, Johannes Kepler University Linz, Altenberger Straße 69, 4040 Linz, Austria.

[2]Institute of Physical Chemistry, University of Innsbruck, Innrain 52c, 6020 Innsbruck, Austria

*Corresponding author: elisabeth.leeb@jku.at

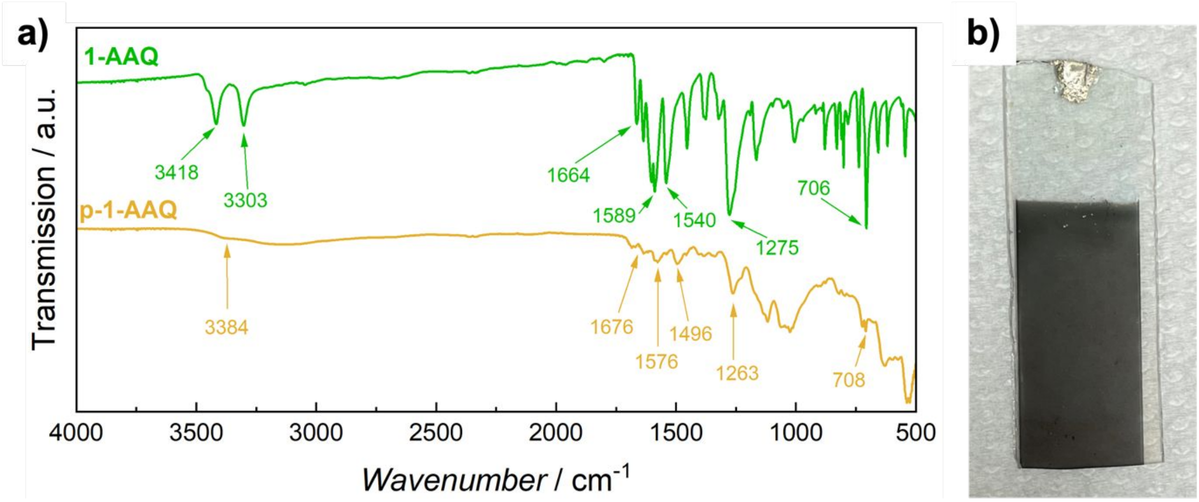

Figure S1: Characterization of polymer with **a)** IR spectrum of monomer 1-AAQ and the resulting polymer p-1-AAQ and **b)** image of p-1-AAQ on a transparent electrode (FTO).

Table S1: Assignment of peaks in the IR spectra to the respective bond vibrations in 1-AAQ and p-1-AAQ[1–3].

**Bonds**	**Wavenumber / cm^{-1}**	
	**1-AAQ**	**p-1-AAQ**
NH-stretch (primary amine)	3418<br>3303	
NH-stretch (secondary amine)		3384 (very broad)
C=O stretching vibration	1664	1676
Stretching vibration of quinoid / benzenoid	1589<br>1540	1576<br>1496
CN-stretch	1275	1263
CH-bend	706	708

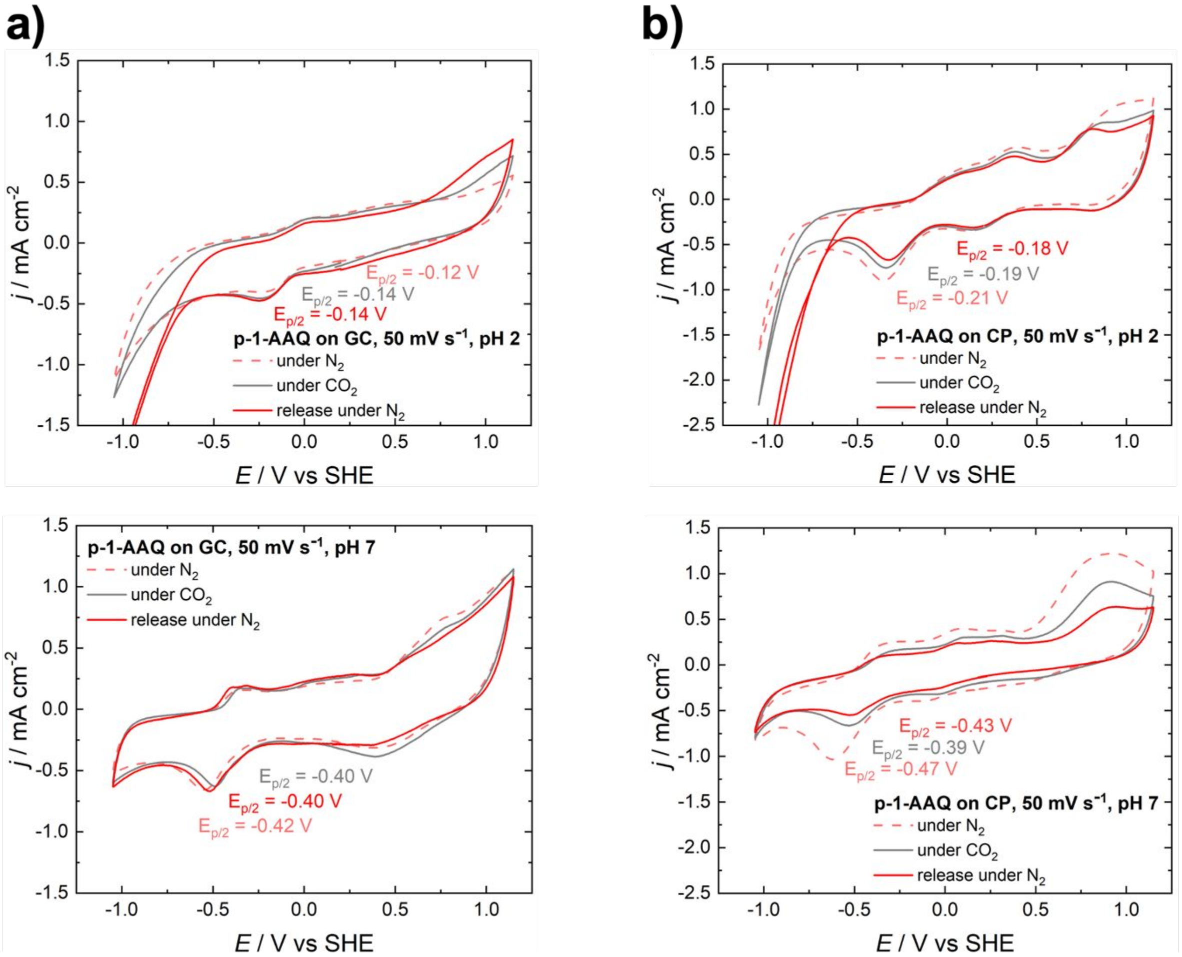

Figure S2: CVs of p-1-AAQ-coated electrodes in a phosphate buffer solution at pH 2 and 7 at a scan rate of 50 mV s^{-1} using a **a)** glassy carbon and **b)** carbon paper electrode.

Table S2: Moles of CO_2 captured as well as faradaic efficiency for the carbon capture using p-1-AAQ-coated glassy carbon and carbon paper electrodes under acidic, neutral and alkaline conditions.

	Cyclic Voltammetry		Electroswing	
**Electrode material**	**n / µmol**	**FE / %**	**n / µmol**	**FE / %**
		**p-1-AAQ at pH = 2**		
Glassy carbon	1.05	16	1.09	92
Carbon paper	0.58	22	0.42	11
		**p-1-AAQ at pH = 7**		
Glassy carbon	0.97	30	1.38	17
Carbon paper	0.16	5	0.25	8
		**p-1-AAQ at pH = 11**		
Glassy carbon	1.24	47	1.44	77
Carbon paper	0.35	14	1.39	31

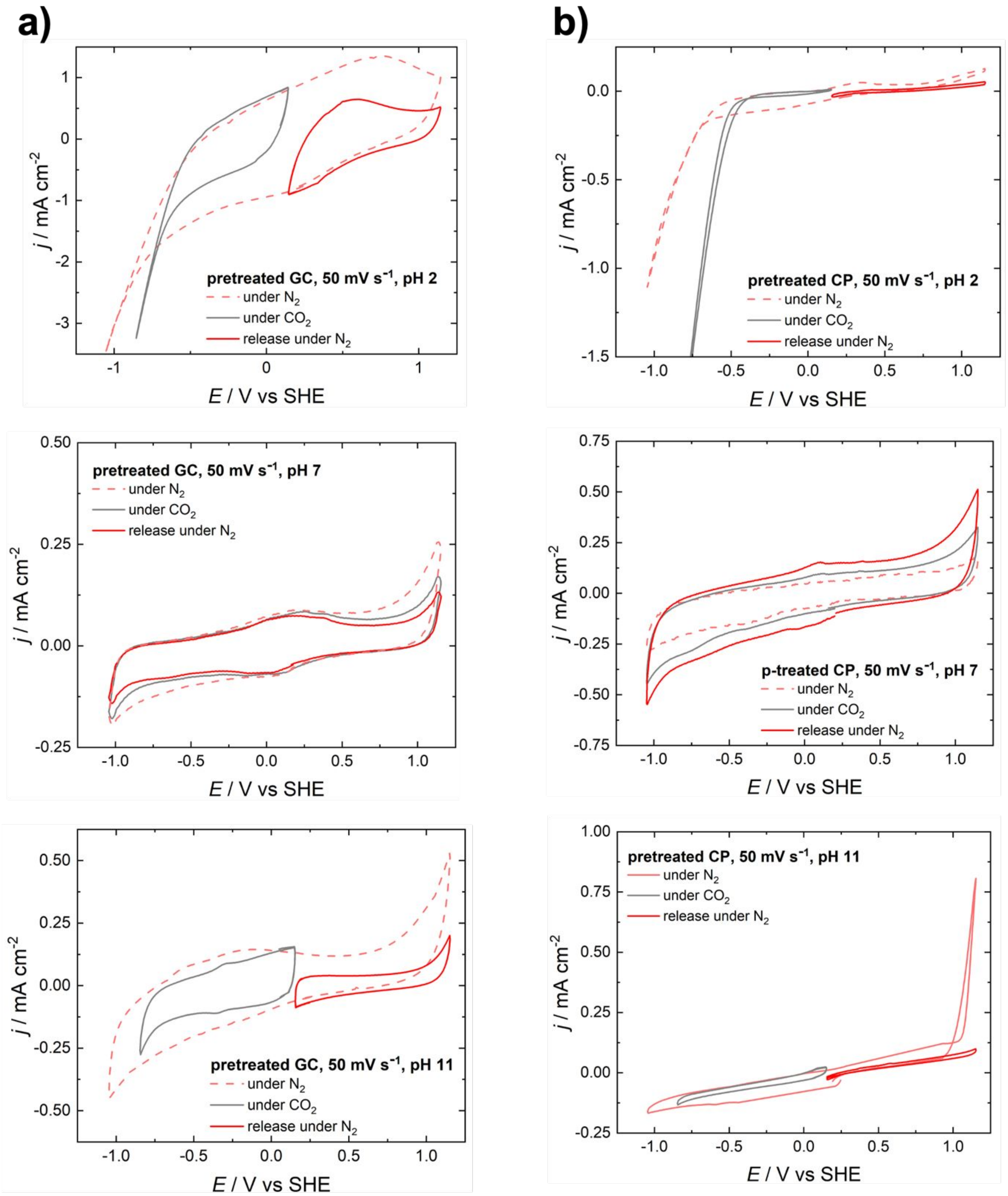

Figure S3: CVs of pretreated electrodes in a phosphate buffer solution at pH 2, 7, and 11 at a scan rate of 50 mV s^{-1} using a **a)** glassy carbon and **b)** carbon paper electrode.

Table S3: Moles of CO_2 captured as well as faradaic efficiency for the carbon capture using a pretreated glassy carbon and carbon paper electrodes under acidic, neutral, and alkaline conditions. Red-highlighted values are outside of the calibration curve.

	Cyclic Voltammetry		Electroswing	
**Electrode material**	**n / µmol**	**FE / %**	**n / µmol**	**FE / %**
		**pretreated at pH = 2**		
Glassy carbon	0.03	1	0.11	2
Carbon paper	0.00	0	0.00	0
		**pretreated at pH = 7**		
Glassy carbon	0.15	8	0.00	0
Carbon paper	0.07	5	0.00	0
		**pretreated at pH = 11**		
Glassy carbon	0.07	2	0.00	0
Carbon paper	0.04	1	0.10	4

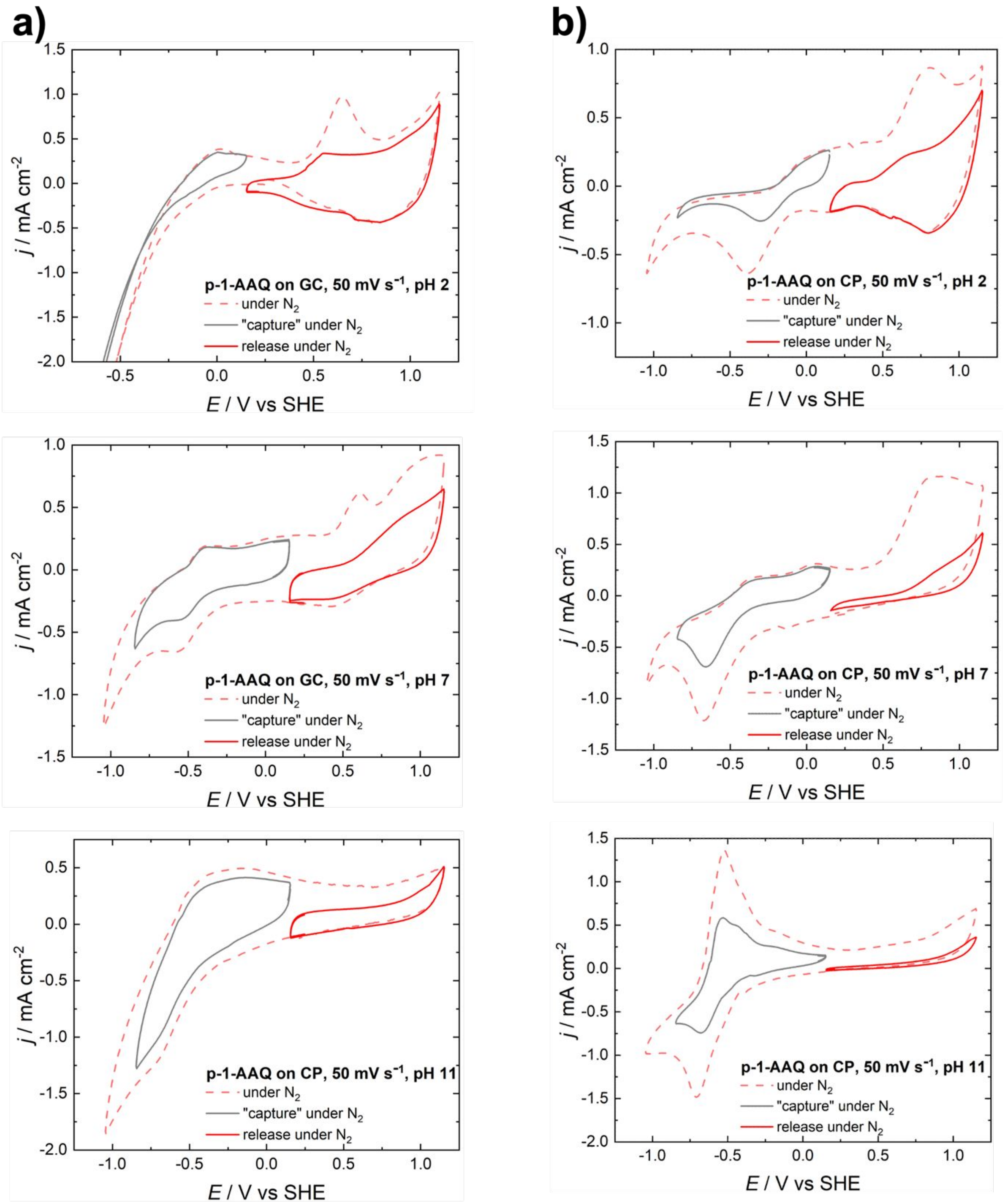

Figure S4: CVs of p-1-AAQ-coated electrodes without CO_2 addition in a phosphate buffer solution at pH 2, 7, and 11 at a scan rate of 50 mV s^{-1} using a **a)** glassy carbon and **b)** carbon paper electrode.

Table S4: Moles of CO_2 captured as well as faradaic efficiency for the carbon capture using a p-1-AAQ-coated glassy carbon and carbon paper electrodes under acidic, neutral, and alkaline conditions without any addition of CO_2. Red-highlighted values are outside of the calibration curve.

	Cyclic Voltammetry		Electroswing	
**Electrode material**	**n / µmol**	**FE / %**	**n / µmol**	**FE / %**
		**without CO_2 at pH = 2**		
Glassy carbon	0.04	1	0.02	0
Carbon paper	0.03	8	0.00	0
		**without CO_2 at pH = 7**		
Glassy carbon	0.00	0	0.00	0
Carbon paper	0.00	0	0.02	2
		**without CO_2 at pH = 11**		
Glassy carbon	0.00	0	0.00	0
Carbon paper	0.00	0	0.04	4

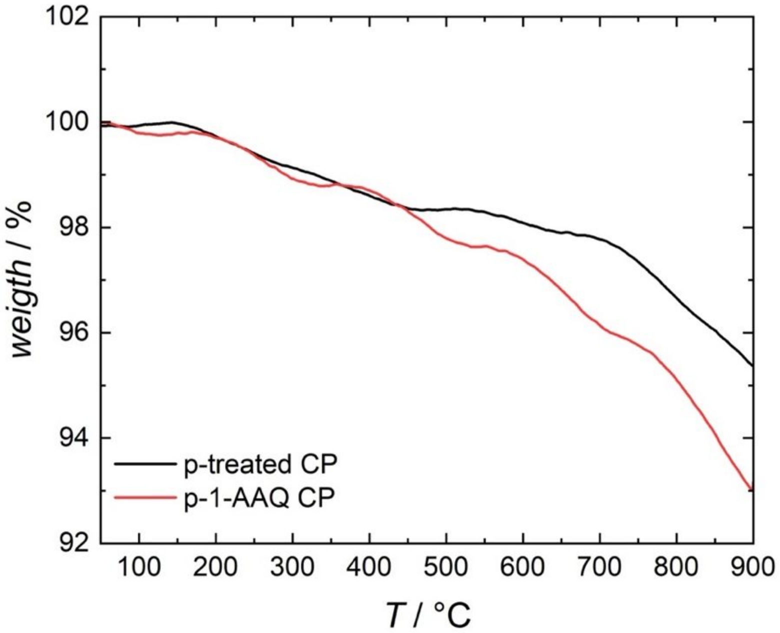

Figure S5: TGA analysis of p-1-AAQ on carbon paper, at a heating rate of 10 °C min^{-1}.

Table S5 Fitting Parameters at different potentials and corresponding mean square deviation for fitting results of p-1-AAQ-coated electrodes in a Ar saturated phosphate buffer solution at pH 11 using a glassy carbon electrode. Fitting was by EC-lab Software V 11.21 using a Randomize+Simplex approach with a weighed Z.

**Pot.**	R_{CF}	R_{dl}	C_{dl}[a]	C_{dlP}	R_p	R_d	t_d	X² / IZI
**V**	Ω	Ω	F (10^{-3})	F (10^{-3})	Ω	Ω	s	
**0.7**	3.5	779	307.0	0.47	15029	16632	100	1.50E-02
**0.4**	3.6	447	277.0	0.51	1921	324734	126	8.94E-03
**0.2**	3.6	167	84.0	0.54	1581	302344	173	8.27E-03
**0.0**	3.6	179	21.4	0.62	2874	148766	29	1.00E-02
**-0.2**	3.6	181	10.9	0.85	1011	179673	213	4.68E-03
**-0.4**	3.6	153	10.0	1.11	556	103312	656	4.37E-03
**-0.6**	3.6	133	7.7	2.03	907	314	79	7.52E-03
**-0.9**	3.7	379	6.1	1.53	3990	2046	87	2.40E-02

[a] Calculated pseudo-capacitance associated with the constant phase element CPE_{dl}^{e}.

Table S6 Fitting Parameters at different potentials and corresponding mean square deviation for fitting results of p-1-AAQ-coated electrodes in a CO_2 saturated phosphate buffer solution at pH 11 using a glassy carbon electrode. Fitting was by EC-lab Software V 11.21 using a Randomize+Simplex approach with a weighed Z.

Pot.	R_{CF}	R_{dl}	C_{dl}[a]	C_{dlP}	R_p	R_d	t_d	X² / IZI
V	Ω	Ω	F (10^{-3})	F (10^{-3})	Ω	Ω	s	
**0.7**	4.5	474	0.5	0.47	3174	16336	81	4.38E-03
**0.4**	4.3	488	1.6	0.31	1094	179173	322	9.65E-03
**0.2**	4.4	771	3.4	0.42	3063	332497	384	6.93E-03
**0.0**	4.4	425	7.1	0.89	1892	860362	5227	2.56E-03
**-0.2**	4.4	493	7.1	1.43	11868	130396	868	5.08E-03
**-0.4**	4.3	983	7.7	2.12	11165	49600	1278	5.63E-03
**-0.6**	4.4	716	3.2	2.89	3365	4324	76	1.24E-02
**-0.9**	4.5	1367	1.0	0.46	12099	12072	120	2.07E-02

[a] Calculated pseudo-capacitance associated with the constant phase element CPE_{dl}^e.

Table S7 Fitting Parameters at different potentials and corresponding mean square deviation for fitting results of p-1-AAQ-coated electrodes in a Ar saturated phosphate buffer solution at pH 11 using a carbon paper electrode. Fitting was by EC-lab Software V 11.21 using a Randomize+Simplex approach with a weighed Z.

Pot.	R_{CF}	R_{dl}	C_{dl}[a]	C_{dlP}	R_p	R_d	t_d	X² / IZI
V	Ω	Ω	F (10^{-3})	F (10^{-3})	Ω	Ω	s	
**0.7**	11.5	612	21.5	0.38	10270	20026	77	2.70E-02
**0.4**	10.8	562	369.0	0.33	732	226421	191	7.25E-03
**0.2**	10.8	491	394.0	0.30	101	318816	189	4.48E-03
**0.0**	10.9	489	177.0	0.29	96	354256	188	3.13E-03
**-0.2**	10.9	110	15.7	0.32	380	86564	92	5.86E-03
**-0.4**	11.4	1001	2.2	2.53	2741	1502	98	8.27E-03
**-0.6**	11.5	849	3.8	4.07	69	7	1	1.10E-02
**-0.9**	11.3	332	12.1	4.82	34	2	3	1.90E-02

[a] Calculated pseudo-capacitance associated with the constant phase element CPE_{dl}^e.

Table S8 Fitting Parameters at different potentials and corresponding mean square deviation for fitting results of p-1-AAQ-coated electrodes in a CO_2 saturated phosphate buffer solution at pH 11 using a carbonpaper electrode. Fitting was by EC-lab Software V 11.21 using a Randomize+Simplex approach with a weighed Z.

Pot.	R_{CF}	R_{dl}	C_{dl}[a]	C_{dlP}	R_p	R_d	t_d	X² / IZI
V	Ω	Ω	F (10^{-3})	F (10^{-3})	Ω	Ω	s	
**0.7**	12.4	239	2.3	0.39	2233	21289	204	7.35E-03
**0.4**	12.7	1407	0.9	0.62	2282	117802	191	2.36E-02
**0.2**	12.6	1063	1.0	0.61	1754	98534	233	2.20E-02
**0.0**	12.2	890	8.4	0.56	912	29667	268	7.30E-03
**-0.2**	12.5	897	12.2	1.70	1747	12615	560	2.80E-02
**-0.4**	12.3	956	7.2	1.88	1852	9177	646	1.07E-02
**-0.6**	12.4	1099	2.4	2.24	1155	6779	818	1.80E-02
**-0.9**	12.6	1923	1.0	0.81	2340	1422	44.9	1.70E-02

[a] Calculated pseudo-capacitance associated with the constant phase element CPE_{dl}^e.

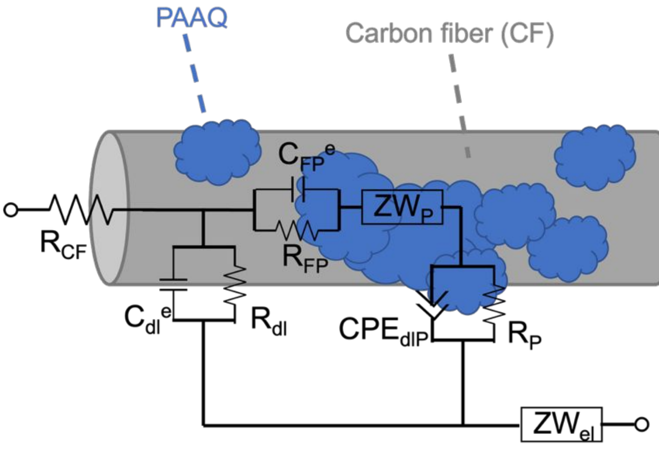

Figure S6: Schematic drawing highlighting the initial best guess for the physical meaning of all of electronic elements used for fitting the measured PEIS data.

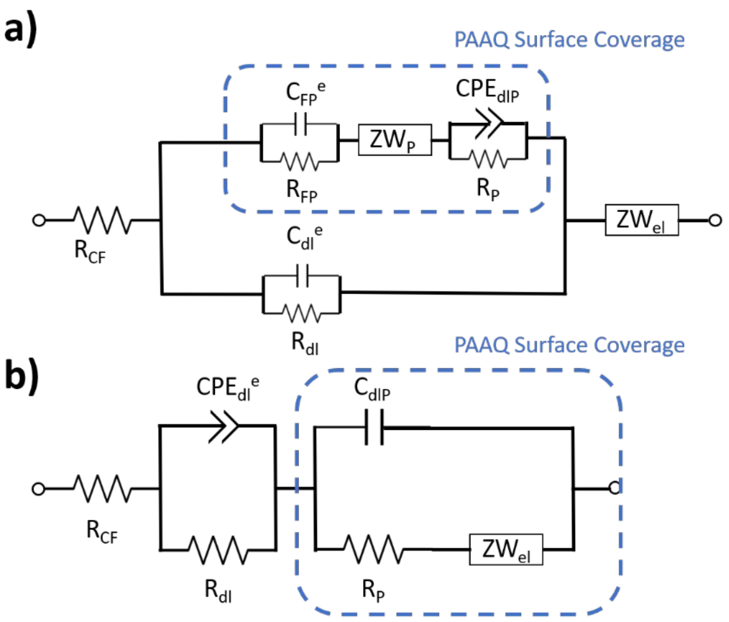

c)

Element	Description
R_{CF}	Ohmic resistance of the carbon support and electrolyte
C_{dl}^{e}	Double layer capacity from the carbon support to the electrolyte
R_{dl}	Double layer resistance from the carbon support to the electrolyte
$C_{FP}e$	Interface capacitance between the carbon support and the PAAQ
R_{FP}	Interface resistance between the carbon support and the PAAQ
ZW_{P}	Ionic and/or electronic diffusion in the PAAQ
CPE_{dlp}	Double layer capacitance between PAAQ and the electrolyte
R_{p}	Double layer resistance between PAAQ and the electrolyte
ZW_{el}	Ionic diffusion in the electrolyte and PAAQ

Figure S7: Equivalent electric circuit used for **a)** initial and **b)** final fitting or the EIS data. **c)** List of electronic elements and corresponding description used within the equivalent electric circuit.

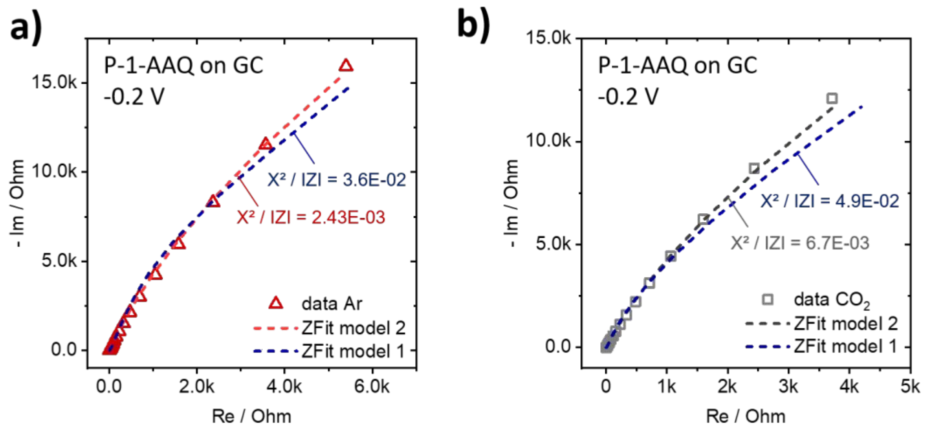

Figure S8: Nyquist plot of the PEIS measurements of p-1-AAQ on GC at pH 7 **a)** under Ar and **b)** CO_2 saturation conditions at a potential of -0.2V. The corresponding fits with the equivalent electric circuit model 1 (**Figure S7a**) and model 2 (**Figure S7b**) are plotted as dashed lines.

Table S9: Fitting Parameters at -0.2 V and corresponding mean square deviation for fitting results of p-1-AAQ-coated electrodes in an Ar and CO_2 saturated phosphate buffer solution at pH 7 using a glassy carbon electrode. Fitting was by EC-lab Software V 11.21 using a Randomize+Simplex approach with a weighed Z. Physically unrealistic values are highlighted by an orange background color.

Pot.	R_{CF}	R_{dl}	C_{dl}[a]	C_{dlP}	R_p	R_d	t_d	X^2 / IZI
V	Ω	Ω	F (10^{-3})	F (10^{-3})	Ω	Ω	s	
**Model 2, Ar**								
**-0.2**	6.9	457	4.85	0.65	1349	134361	202	2.43E-03
**Model 2, CO_2**								
**-0.2**	7.8	441	5.73	0.61	1141	117056	156.9	6.70E-03
**Model 1, Ar**[b]								
**-0.2**	7.1	11256	0.32	-1000.4	1.9	7.7	12E-3	3.60E-2
**Model 1, CO_2**[b]								
**-0.2**	7.5	3.8E27	0.54	31E-24	85.7	192	2.9	4.90E-2

[a] Calculated pseudo-capacitance associated with the constant phase element CPE_{dl}^{e}.

[b] Additional values obtained for the more complex Model 1, Ar: R_{FP} and C_{FP} are 167 288 Ω and 1.26E-3 F, respectively and ZW_P with 388.9 Ω·$s^{-1/}$; Model 1, CO_2: R_{FP} and C_{FP} are 3.5 Ω and 1.715 F, respectively and ZW_P with 5 078 Ω·$s^{-1/}$;

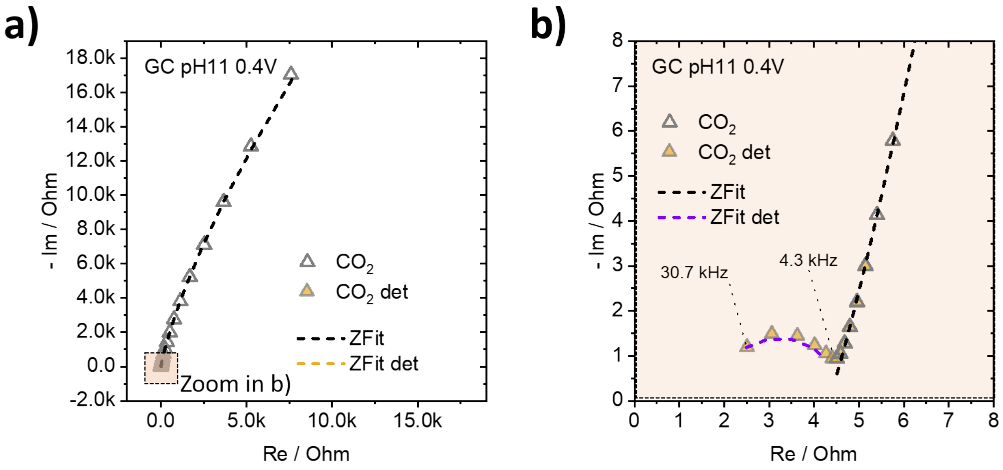

Figure S9: Nyquist plot of the PEIS measurements of p-1-AAQ on GC at pH 11 and under CO_2 saturation conditions showing the appearance of an additional impedance signal at high frequencies, between 30.7 kHz and 4.3 kHz (b). Symbols represent the experimental data and the lines are the best fit.

(1) Gao, M.; Yang, F.; Wang, X.; Zhang, G.; Liu, L. Electrochemical Characteristics and Stability of Poly(1,5-Diaminoanthraquinone) in Acidic Aqueous Solution. *J Phys Chem C* **2007**, *111* (46), 17268–17274. https://doi.org/10.1021/jp074415j.

(2) Li, X. G.; Li, H.; Huang, M. R.; Moloney, M. G. Synthesis and Multifunctionality of Self-Stabilized Poly(Aminoanthraquinone) Nanofibrils. *J Phys Chem C* **2011**, *115* (19), 9486–9497. https://doi.org/10.1021/jp201967n.

(3) Ding, Y.; Ren, X.; Chen, D.; Wen, F.; Li, T.; Xu, F. Poly(1,5-Diaminoanthraquinone) as a High-Capacity Bipolar Cathode for Rechargeable Magnesium Batteries. *ACS Appl Energy Mater* **2022**, *5* (3), 3004–3012. https://doi.org/10.1021/acsaem.1c03652.
